# Lymph Node Assessment with Multiparametric Ultrasound: Normal Values, Morphologic Patterns, and Diagnostic Algorithms

**DOI:** 10.3390/cancers18061045

**Published:** 2026-03-23

**Authors:** Kathleen Möller, Christian Jenssen, Markus Herbert Lerchbaumer, Alois Hollerweger, Madhvi Yadav, Manjiri Dighe, Carla Serra, Andrea Boccatonda, Siegbert Faiss, Christoph Frank Dietrich

**Affiliations:** 1 Medical Department I/Gastroenterology, SANA Hospital Lichtenberg, 10365 Berlin, Germany; k.moeller@live.de (K.M.); siegbert.faiss@sana.de (S.F.); 2 Department of Internal Medicine, Krankenhaus Märkisch Oderland, 15344 Strausberg, Germany; c.jenssen@khmol.de; 3 Brandenburg Institute for Clinical Ultrasound (BICUS), Brandenburg Medical University, 16816 Neuruppin, Germany; 4 Department of Radiology, Charité-Universitätsmedizin Berlin, Charitéplatz 1, 10117 Berlin, Germany; markus.lerchbaumer@charite.de; 5 Department of Radiology, Hospital Barmherzige Brüder, 5020 Salzburg, Austria; alois.hollerweger@aon.at; 6 Department of Radiology, University of Washington, Seattle, WA 98195, USA; madhvi@uw.edu (M.Y.); dighe@uw.edu (M.D.); 7 Diagnostic and Therapeutic Interventional Ultrasound Unit, IRCCS Azienda Ospedaliero-Universitaria di Bologna, Policlinico Sant’Orsola-Malpighi, Via Massarenti N 9, 40138 Bologna, Italy; carla.serra@aosp.bo.it (C.S.); andrea.boccatonda2@unibo.it (A.B.); 8 Center of Excellence and Education, Haldenweg 11A, 3626 Thun, Switzerland

**Keywords:** lymph nodes, reference values, transcutaneous ultrasound, multiparametric ultrasound

## Abstract

Background/Objectives: Transcutaneous ultrasound (US) is the first-line imaging modality for detecting and characterizing lymph nodes (LNs), enabling further lesion discrimination regarding potential malignancy. Accurate interpretation requires both knowledge of normal reference values and a multiparametric diagnostic approach. Methods: This narrative review was based on a comprehensive literature review. Results: The article summarizes current evidence on normal LN morphology and measurements in B-mode US, color Doppler imaging (CDI), elastography, and contrast-enhanced ultrasound (CEUS). Typical sonomorphologic features of benign and malignant LNs, including size, shape, echogenicity, vascular patterns, stiffness, and perfusion characteristics, are presented alongside corresponding reference values from published studies. The diagnostic limitations and overlaps between normal, inflammatory, and malignant LNs are highlighted, emphasizing potential pitfalls in interpretation. Multiparametric ultrasound combining B-mode, CDI, elastography, and CEUS enhances diagnostic confidence, reduces the need for invasive procedures, and supports standardized LN assessment in clinical and oncologic practice. Conclusions: This article is part of a series on normal reference values in US imaging. Knowledge of normal values and integration of multiparametric findings form the basis for accurate LN characterization and reduce the need for invasive diagnostics.

## Simple Summary

Lymph nodes (LNs) are an essential part of the immune system and are frequently evaluated when infection, inflammation, or cancer is suspected. Ultrasound is the preferred first-line imaging method for LN assessment because it is non-invasive, widely available, and does not involve radiation. Accurate interpretation, however, requires knowledge of normal LN appearance and an understanding of how different disease processes may alter ultrasound findings. This article summarizes established normal values and characteristic morphologic and functional patterns of LNs using multiple ultrasound techniques. The authors aim to provide practical guidance for differentiating benign from malignant LNs in routine clinical practice. Improved standardization and multiparametric assessment may increase diagnostic confidence, reduce unnecessary invasive procedures, and support more consistent LN evaluation in oncologic and general medical settings.

## 1. Introduction

There are various scenarios that require a differentiated assessment of enlarged lymph nodes (LNs), including, in particular, focal swelling or known malignancies. Understanding the normal LN morphology and reference values is essential for differentiating benign from malignant pathology. Abnormal LNs must always be assessed in the overall context of the patient’s medical history, clinical complaints, and symptoms. If there is inflammation in an anatomical region, there is a high probability that LN is also inflammatory in origin. However, if a history of a malignant tumor is known, it is primarily assumed that LN is metastatic. Furthermore, there are enlarged LNs that are conspicuous to the US but cannot be classified and must ultimately be specified by histological confirmation.

The aim of ultrasound (US) diagnostics is to evaluate criteria for distinguishing the possible causes of LN enlargement and to limit the need for histological confirmation or radiation-exposing imaging modalities such as Positron emission tomography–computed tomography (PET-CT) to absolutely necessary indications.

Sonographic assessment of LNs is performed either as an integral part of the assessment of an anatomical region or specifically as part of tumor staging. The US appearance changes both in inflammatory diseases and in LN metastases and malignant lymphomas. In malignant diseases, the assessment of nodal malignancy is used to classify the N stage and integrated into oncological follow-up. Especially rare diseases like tuberculous lymphadenitis or sarcoidosis present a diagnostic problem and can often be difficult to distinguish from malignant lymphadenopathy. Reactive or post-inflammatory benign LN enlargement, on the other hand, may have a different appearance than completely inconspicuous regional LNs in asymptomatic subjects. US remains the primary imaging modality used to evaluate LN stations. The aim is to detect enlarged LNs, differentiate between regional unremarkable LNs and pathological LNs, and distinguish between inflammatory and malignant LNs.

Superficial LNs, in particular, are easily accessible by US. Examination with high-resolution linear transducers is the method of choice. The assessment of LN structures in the abdominal and retroperitoneal areas depends on the individual sonographic accessibility of the patient. However, LN structures are visible in a large number of patients, especially in non-obese patients or those with enlarged LNs. It is reported that up to 55% of healthy subjects had distinguishable LNs in various abdominal parts [1].

Based on publications by Sienz et al. [2,3,4] on normal values in ultrasound of the hepatobiliary system, spleen, kidneys, and abdominal vessels, this article is a further contribution to a series on the normal values in ultrasound and their significance [5,6,7,8,9,10,11,12,13,14]. Depending on the organ system, this series considers ethnic characteristics, age, gender, constitution, or a multiparametric approach.

## 2. Method

**Figure 1:**
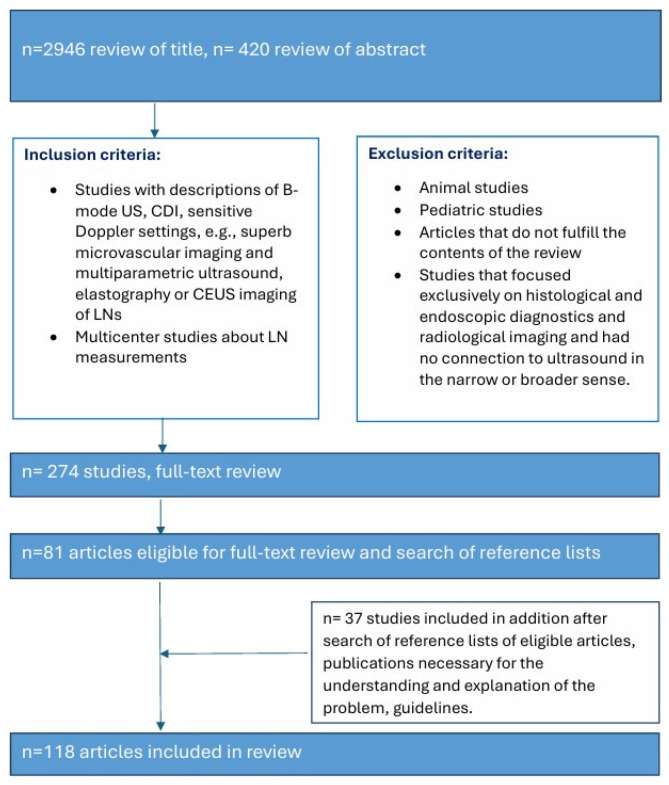
Search strategy.

The PubMed Database was comprehensively searched and screened for eligible studies up to 30 September 2025. Terms related to reference values of LNs in B-mode ultrasound, color Doppler imaging, and elastography were used as keywords. The keywords and binary operators are listed in brackets: Lymph nodes AND (ultrasound OR reference values OR normative values OR standard values OR short-axis measurement). Lymph nodes AND (vascularity OR color Doppler imaging OR Power Doppler Ultrasonography OR resistive index OR pulsatility index OR vascular pattern). Lymph nodes AND (CEUS OR superb microvascular imaging OR multiparametric ultrasound). Lymph nodes AND elastography. Lymph nodes AND (US-guided biopsy). The literature was complemented by extensive cross-checking of the reference list of the retrieved articles [Figure 1].

Histopathologic correlation remains the reference standard for lymph node characterization. As this article focuses on normal reference values and sonomorphologic patterns rather than quantitative diagnostic accuracy, reported sensitivity and specificity vary considerably across studies, anatomical regions, and underlying diseases. Where available, studies with histological or cytological confirmation are explicitly indicated, and the overlap and limitations of individual ultrasound criteria are emphasized. Multiparametric ultrasound should, therefore, be understood as a tool to improve pre-test probability, support clinical decision-making, and guide targeted biopsy, rather than as a replacement for histopathologic diagnosis.

### 2.1. Investigation Requirements

The examination of superficial LNs should be performed using high-frequency linear US probes (9–18 MHz). A sensitive Doppler preset, including low pulse repetition frequency and optimized Doppler gain settings, should be used to enhance visualization of low-flow vascular structures in superficial LN assessment.

In the abdominal and retroperitoneal areas, a curved array transducer with medium and higher frequencies (1–6 MHz) should be available, as well as high-frequency linear transducers with medium frequencies (2–9 MHz). For a multiparametric US approach (mpUS), contrast-enhanced ultrasound (CEUS) should be available, as well as the microvascular imaging technique, strain elastography (SE), or shear wave elastography (SWE) for superficial LNs.

Superficial LNs can be examined at any time. For the scheduled examination of abdominal and retroperitoneal LNs, it is recommended that patients fast for at least four hours to reduce the amount of food and air in the small bowel [15] and improve sonographic visibility.

### 2.2. Lymph Node Stations

**Figure 2:**
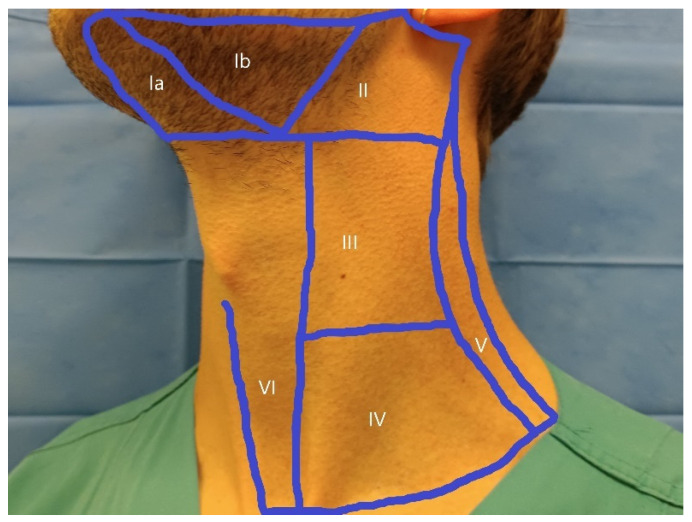
Cervical LN regions according to Som [15]: Ia—submental region, Ib—submandibular region, II—upper, III—middle, and IV—lower cervical region along the large vessels, V—posterior cervical triangle, VI—pre- and paralaryngeal region.

The superficial LN stations include those in the head and neck region and the axillary and inguinal LNs. Infraclavicular LNs can also be viewed. In the head and neck, the following levels are classified according to Som [16]: Ia—submental region, Ib—submandibular region, II—upper jugular, III—middle jugular, and IV—lower jugular region along the large cervical vessels, V—posterior cervical triangle, VI—pre- and paralaryngeal region [Figure 2].

**Figure 3:**
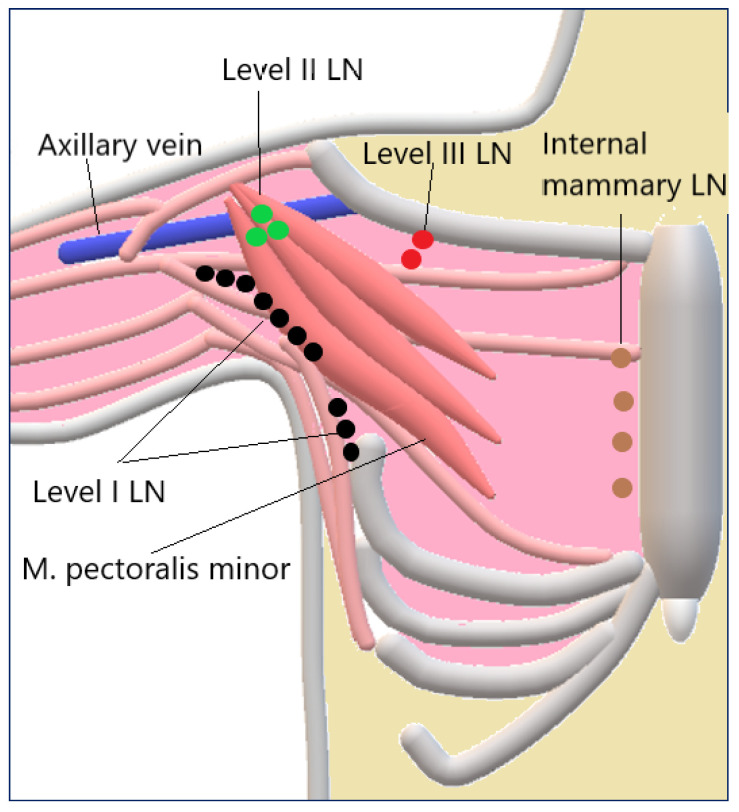
Axillary, level I–III, and internal mammary LNs schematically illustrated.

The left supraclavicular region warrants special attention because involvement of Virchow’s LN is a known indicator of potential malignancy. The axillary LN are divided into three levels depending on their position relative to the pectoralis minor muscle. Level I is located caudally and laterally to the pectoralis minor muscle. Level II is located between the lateral and medial edges of the pectoralis minor muscle. Level III is located cranially and medially to the pectoralis minor muscle. The various axillary LN stations are important in the staging of breast cancer [Figure 3].

The internal mammary LNs are located parasternally along the internal mammary vessels. Ultrasound plays a significant role as a primary imaging modality and also has multiparametric value for patient stratification in breast cancer.

Axillary ultrasound can reduce the need for sentinel LN biopsy in selected patients. However, its diagnostic accuracy depends on the examiner’s experience and standardized criteria.

In the abdomen and retroperitoneum, Dietrich et al. examined the LNs associated with the hepatoduodenal ligament: ventral to the portal vein next to the common hepatic artery and between the portal vein and the inferior vena cava [17].

Additional LN localizations have been evaluated as well, including the LNs interaortocaval, peripancreatic, right mesenteric, and along the renal vessels [1]. In addition, complex or targeted sonographic examinations also include paraaortal, paracaval, and iliac LNs and interenteric or paracolic LNs. The sonographic assessment of the intestine includes the assessment of the corresponding mesenteric LN stations.

Transcutaneous imaging of mediastinal LNs includes supraaortic, paratracheal, aortopulmonary, prevascular, subcarinal, and pericardial LN stations [18]. Of note, mediastinal LNs are easier assessed using computed tomographic imaging or, depending on their localization, in transesophageal or endobronchial endosonography (EUS, EBUS). Transcutaneous detection requires a high level of expertise and intrinsic motivation [18].

The assessment of paraesophageal, paragastric, paraduodenal, and pararectal LNs in tumor staging is a domain of EUS.

The LNs in the extremities are divided into superficial and deep LNs. This also includes the aforementioned axillary LN stations. Further deep arm LNs are found along the medial side of the brachial artery, as well as in the cubital fossa, in the bifurcation of the brachial artery (known as the deep cubital node), and occasionally along the arteries of the forearm. Other superficial arm LNs are located along the superficial veins. Superficial cubital/supratrochlear nodes are located 5 cm above the medial epicondyle along the basilic vein. There are a few LNs in the popliteal fossa that drain the foot, lower leg, and knee. For more information on the LNs in the extremities, please refer to the relevant literature [19,20,21,22].

The choice of transducer and examination technique for LNs is summarized in Table 1.

### 2.3. Lymph Node Sonomorphology

#### 2.3.1. What Is Normal (Reference Values)

**Figure 4:**
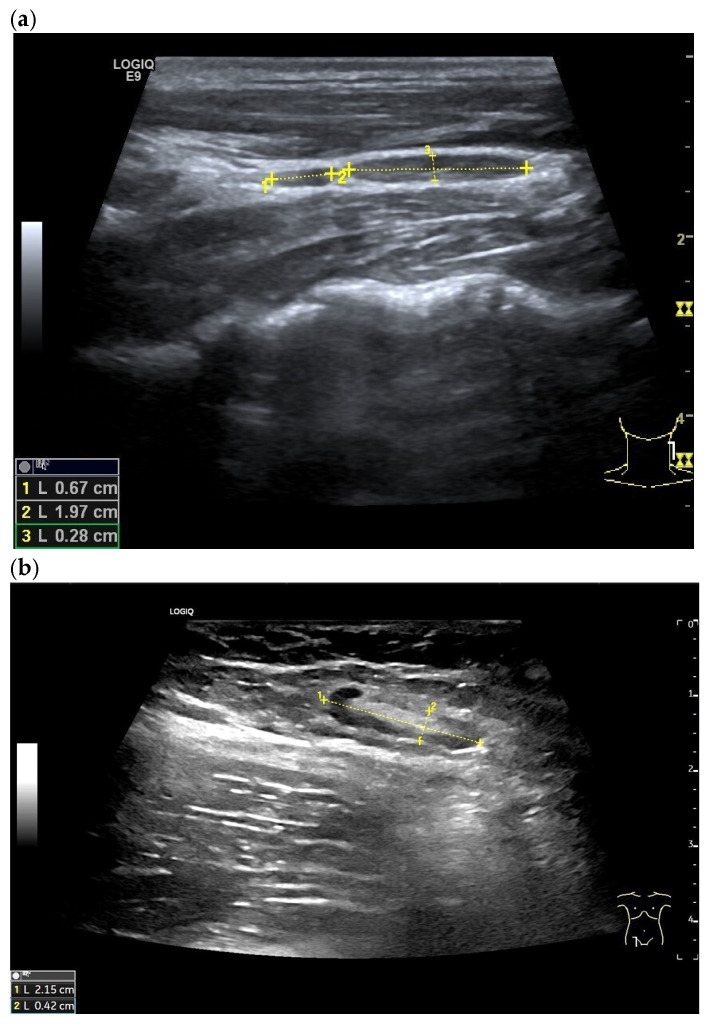

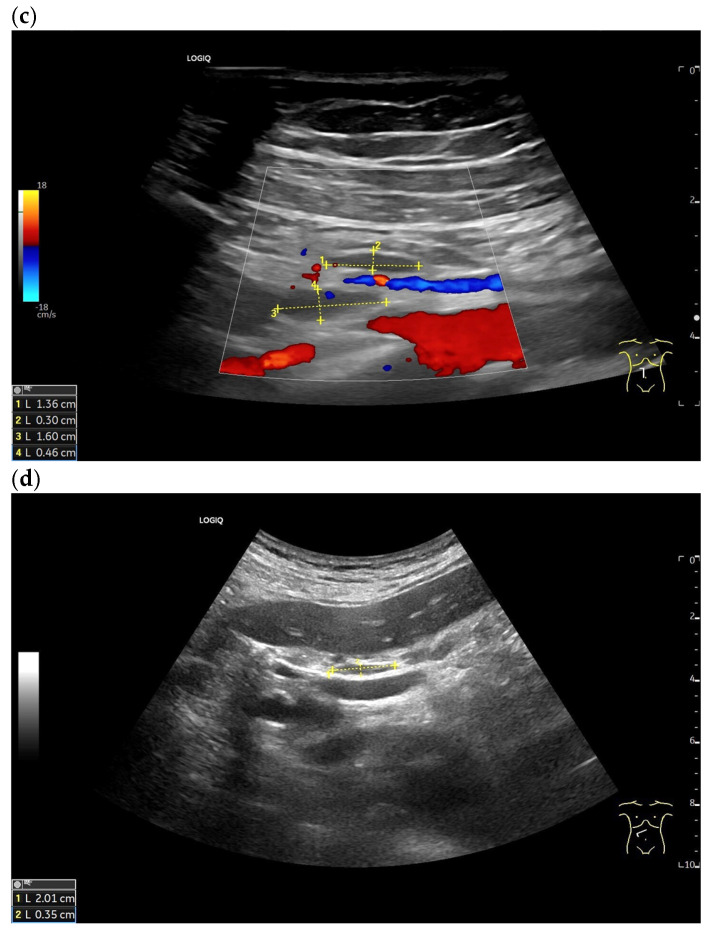
Normal regional LNs in healthy subjects (between the markers): cervical (**a**), inguinal (**b**), paraaortic (**c**), and hepatic hilar (**d**). The LNs have an elongated oval shape. The short-axis is < 5 mm. Only the inguinal LN (**b**) has a distinct hyperechoic hilum.

**Figure 5:**
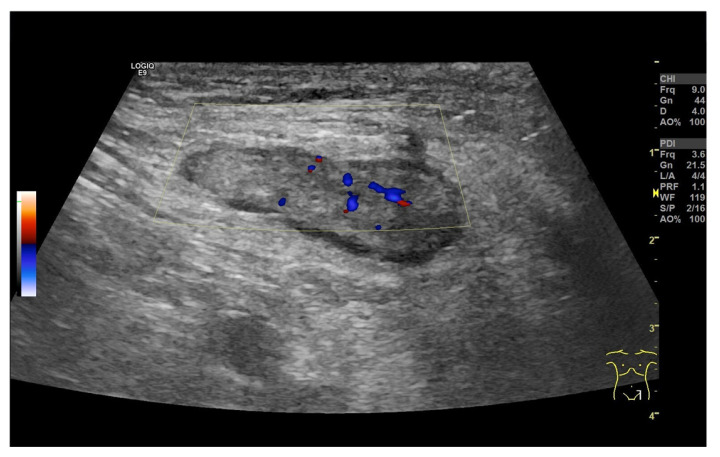
Inguinal LN, fatty involuted with broad hyperechoic center and very narrow hypoechoic cortex.

A normal LN has an elongated oval shape [Figure 4]. The LN hilum is located in the center, with connective tissue and fatty tissue. A central afferent arterial vessel, accompanied by the efferent vein, leads into the LN. The LN hilum is usually hyperechoic due to numerous interface artifacts. Sometimes, a delicate hypoechoic layer corresponding to pure fatty tissue can be distinguished centrally around the echogenic hilum. This is, in turn, separated from the cortex by a delicate fibrovascular band [23]. This hypoechoic band has been described in inguinal or axillary LNs, but not in cervical LNs [23]. Fatty involuted LNs contain a higher proportion of fat and connective tissue in the center [Figure 5]. The hypoechoic cortex of the LN is located at the periphery. The hyperechoic central hilum is regularly present in the inguinal and axillary regions, but may be absent in the neck in stations II, III, IV, and V [23,24,25]. With increasing age, the central hyperechoic reflex can also be distinguished in cervical LNs [25].

The hyperechoic LN hilum is generally considered a sign of benignity. In contrast to physiological axillary and inguinal LNs, many normal cervical LNs do not have a hyperechoic hilum [24,25], possibly due to detection at very small sizes. Typically, metastatic infiltrated LNs in these regions may also present in their early stages with a preserved echogenic hilum.

#### 2.3.2. What Suggests Malignancy

Metastatic processes are initially localized in the hypoechoic cortex and then lead to compression (e.g., excentric hilum) or infiltration with disappearance of the hilum [26].

#### 2.3.3. Overlap and Pitfalls

Nevertheless, the absence of the central hilum in the neck is not necessarily associated with tumor manifestation [27], and the presence of a hyperechoic hilum in other locations does not rule out a metastatic tumor process [28,29].

In the abdomen, the visualization of a present hilum may be limited with convex transducers due to the depth of penetration [1,30].

### 2.4. Size

In accordance with its oval shape, the LN has a longitudinal diameter, depth diameter, and transverse diameter. In imaging, the terms long-axis diameter (e.g., longitudinal diameter) and short-axis diameter are usually specified. In CT and MRI, LNs are often cut diagonally, so that the longitudinal diameter cannot be determined correctly. Therefore, the short-axis is of greater importance on CT and MRI images. The short-axis is also affected by an increase in size as the first.

The size of LNs can vary greatly. Since pathological LNs become rounded as they increase in size, particularly in the short-axis, some authors have placed greater emphasis on the short-axis [31,32,33,34,35].

#### 2.4.1. What Is Normal (Reference Values)

Since the hyperechoic hilum is usually absent in cervical LNs, the short-axis diameter is particularly well suited for use in the neck region. Rettenbacher [23] and other authors recommend a threshold value of 8 mm for the short LN-axis submandibular and in the upper neck region (regions Ib and II) [24,27,31,32,36]. For all other neck regions (Ia, III, IV, and V), this threshold value was defined as 5 mm for benignity [24]. The jugulodigastric LN is located cranial to the bifurcation of the carotid artery, lateral to the great neck vessels. It belongs to region II. It is one of the largest cervical LNs [37].

The long-axis normal value for LNs in the hepatoduodenal ligament is an upper value of 17 mm [17,30]. For LNs in the hepatoduodenal ligament, as well as abdominal and retroperitoneal LNs, the studies by Dietrich et al. indicate a normal short-axis diameter of 5–6 mm [1,17]. This correlates with CT data on the short-axis diameter of mesenteric LNs with a maximum of 5 mm [38].

For axillary and inguinal LNs with a wide central lymph node hilum, Rettenbacher [23] recommends measuring the cortex width. In 95% of normal subjects, the cortex width was no more than 4 mm in axillary LNs and no more than 2.5 mm in inguinal LNs [23].

#### 2.4.2. What Suggests Malignancy

Using oral squamous cell carcinoma as an example, it was shown that the probability of cervical LN metastases increases with size [39]. The histologically positive rates were 25%, 80%, and 93% for nodes between 5 and 10 mm, 10 and 15 mm, and for nodes > 15 mm (without specifying whether short or long-axis), respectively [39].

Lyshchik et al. [35] calculated a significant difference in the short-axis diameter between metastatic (9.1 mm ± 5.8; 95% CI: 7.6 mm ± 10.6 mm) and benign (6.6 mm ± 3.4; 95% CI: 5.8 mm ± 7.4 mm) cervical LNs (*p* < 0.01). No precise information was provided regarding the localizations.

According to RECIST 1.1, lymph nodes are measured in their short-axis. Nodes with a short-axis < 10 mm are considered normal, even in oncology patients. Nodes measuring 10–14 mm are regarded as pathologic but are not measurable as target lesions, whereas nodes ≥ 15 mm are considered pathologically enlarged and eligible as target lesions [40,41].

For response assessment, any pathological node must regress to a short-axis of less than 10 mm to be considered normal again, underscoring that the 10 mm short-axis threshold is the pivotal cut-off separating normal from abnormal lymph node size in RECIST-based imaging studies [40].

#### 2.4.3. Overlap and Pitfalls

In the study of Lyshchik et al. 21% of the benign LNs were larger than 8 mm, while only just under half (47% of the metastatic LNs) were larger than 8 mm (*p* < 0.01) [35].

A size variation range of 1–10 mm was observed in the neck area, for example, but not beyond that [31]. However, if a large proportion of the benign LNs contained tuberculosis, these diameters were also higher [42,43]. Why the mean diameter of non-metastatic, reactive cervical LNs in a study from Iran was 16.3 mm is not explained [33].

Study data on the size of benign LNs are summarized in Table 2 and Table 3. These data refer to healthy subjects as well as to the group of non-malignant LNs in tumor patients. In addition, some studies include patients with tuberculous LNs. In these studies, the values for benign LNs are larger.

Table 4 summarizes the reference values based on the studies. It should be noted that choosing a higher cut-off value results in high specificity but low sensitivity, while choosing a lower cut-off value results in higher sensitivity but lower specificity for the presence of lymphadenopathy. Normal LN sizes vary according to the different anatomic localizations. However, size should never be interpreted as the sole criterion, but always in conjunction with the other B-mode US features.

### 2.5. Solbiati Index and Short/Long-Axis Ratio

**Figure 6:**
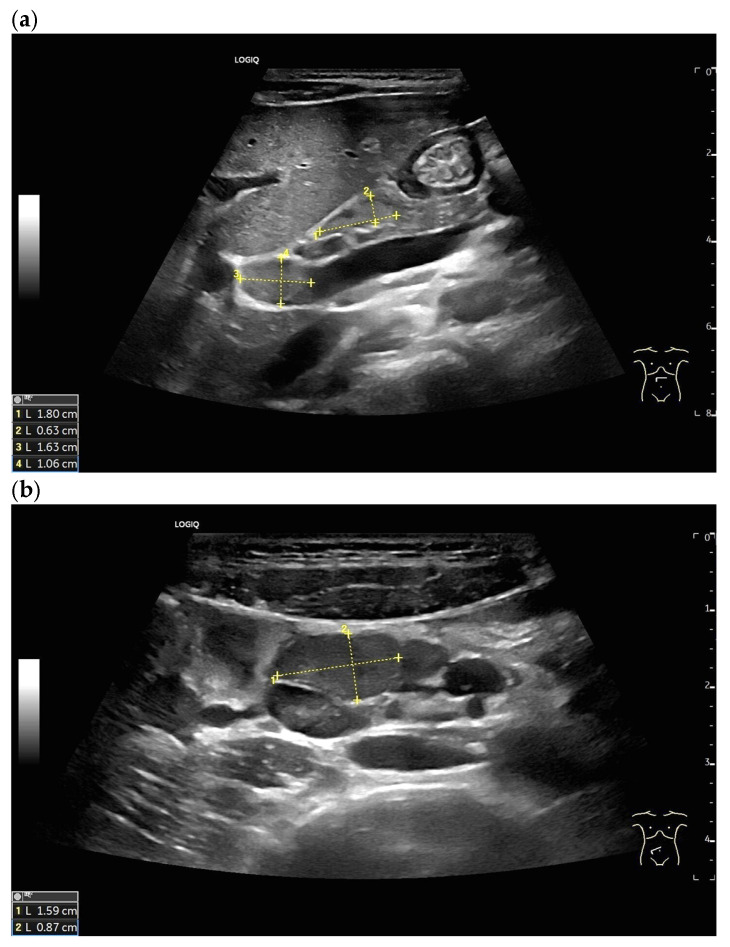
LNs with rounded shape in the hepatic hilum in cases of histologically confirmed overlap syndrome of autoimmune hepatitis and primary sclerosing cholangitis (**a**) and in the mesentery in Crohn’s disease (**b**).

**Figure 7:**
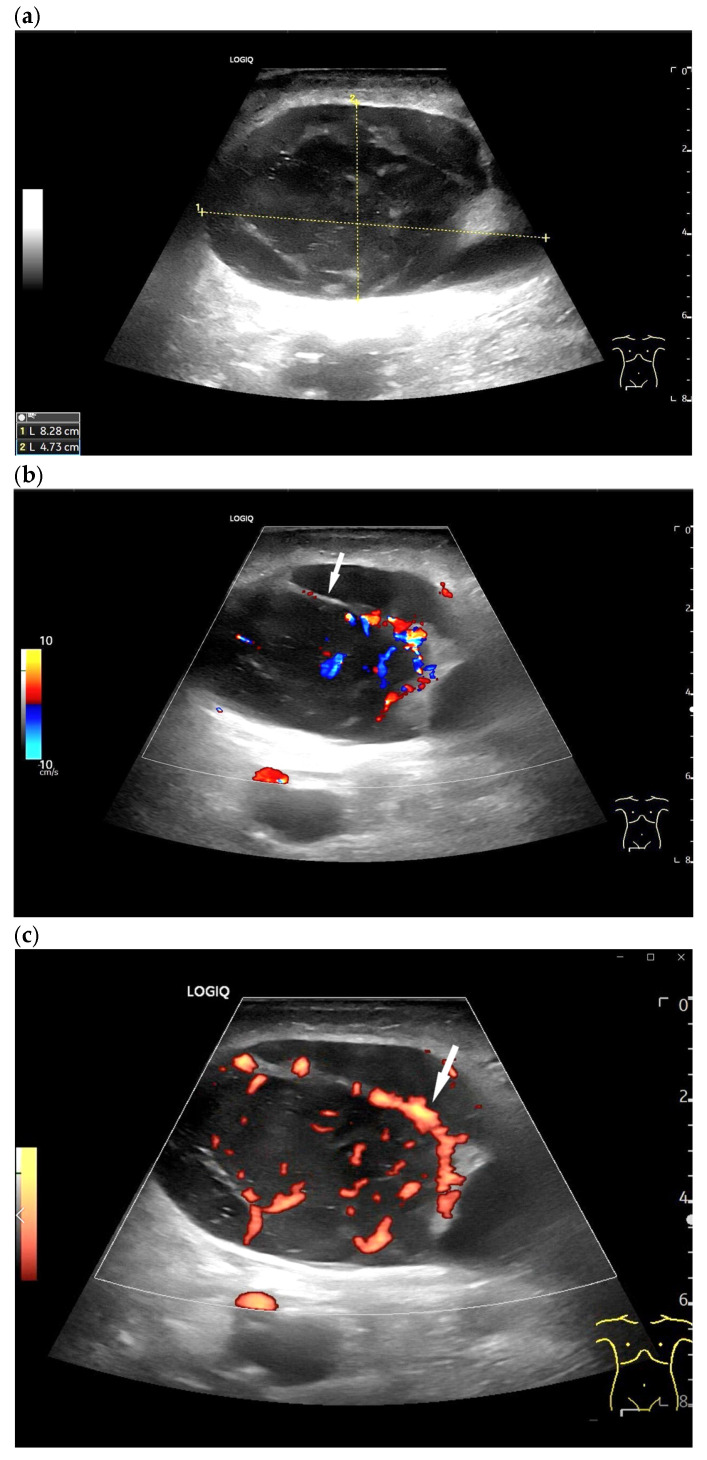
Diffuse large B-cell lymphoma. Pathological LN in the right inguinal region. This appears as a large, round, distinctly hypoechoic mass. Another lesion is visible within the mass (**a**). The LN hilum is narrow and compressed and located at the edge (arrow). A central hilar vessel cannot be distinguished in duplex sonography (**b**). A macro vessel is visible in power Doppler (arrow) (**c**). Individual vascular reflexes are visible next to the hilum. Otherwise, distributed irregular vessels are visible—vascular pattern V. US-guided sampling was performed with a histological diagnosis of diffuse large B-cell lymphoma.

Short-axis enlargement leading to LN rounding is a sonographic feature associated with nodal pathology, including metastatic involvement and malignant lymphoma [26]. However, inflammation, like reactive inflammatory processes [33], sarcoidosis, or tuberculosis, can also lead to rounding of the LNs [Figure 6 and Figure 7].

### 2.6. Solbiati Index

**Figure 8:**
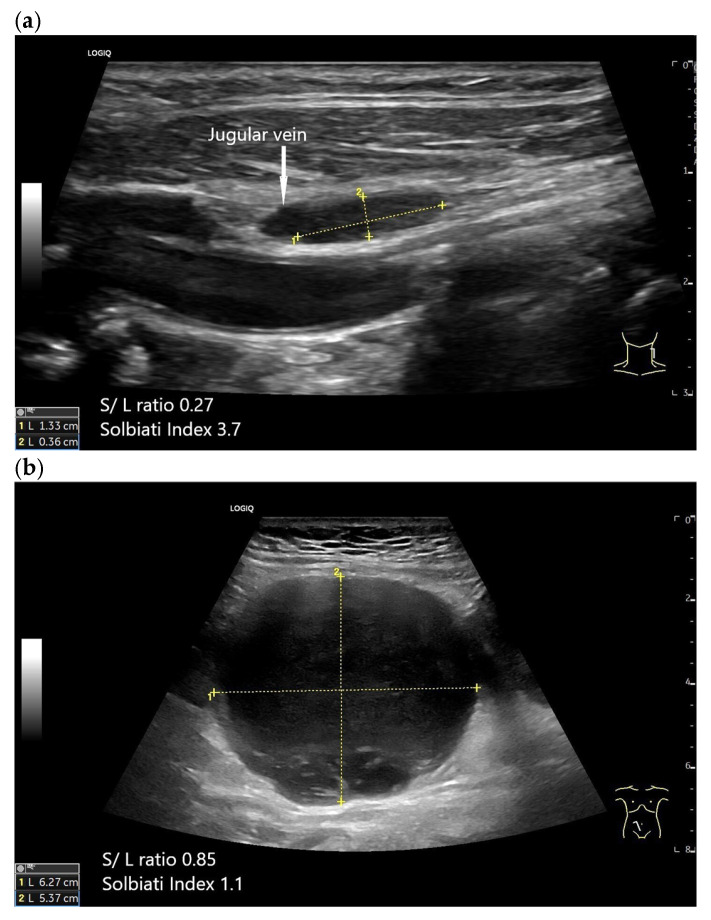
Normal oval-shaped cervical LN with an S/L ratio < 0.5 and a Solbiati index > 2 (**a**). Pathological lymph node in the right inguinal region in a case of diffuse large B-cell lymphoma (**b**).

The Solbiati index describes the ratio of the longitudinal diameter to the short-axis diameter, i.e., the quotient of long-to-short-axis [54]. The Solbiati index is described in particular for assessment of LN metastases in the head and neck region [54], but can also be used for other LN localizations [Figure 8].

#### 2.6.1. What Is Normal (Reference Values)

A Solbiati index above 2 is mainly seen in benign LNs [17]. A Solbiati index > 2 strongly suggests benign behavior but should always be interpreted in context with vascularity and echogenicity.

#### 2.6.2. What Suggests Malignancy

A Solbiati index < 2 and especially < 1.5 is a criterion for malignancy [54].

In oral squamous cell carcinoma, high significance for malignancy was shown for round LNs larger than 9 mm, with a positive rate of 14% for LNs measuring 5–9 mm and 86% for LNs larger than 9 mm [39]. The diameter of the short LN-axis should be less than half of the long-axis [30].

#### 2.6.3. Overlap and Pitfalls

In the study by Chen et al. [42], approximately one-third of the benign LNs corresponded to granulomatous inflammation or tuberculosis. However, of these patients, only 37.5% had a Solbiati index > 2.

In a study with benign and malignant cervical LNs (metastases of regional squamous cell carcinoma, metastases of other carcinomas, chronic lymphocytic leukemia, other lymphomas, and other tumors), the malignant LNs had a larger longitudinal diameter and short-axis diameter than the benign LNs (Table 2) [49]. The Solbiati index in benign cervical LNs was significantly higher compared to malignant ones (2.11 ± 0.81 versus 1.6 ± 0.46, *p* < 0.001) [49]. However, there were malignant LNs with a Solbiati index > 2. In this subgroup, the short-axis diameter was significantly larger in the malignant group than in the benign group (1.12 ± 0.62 cm versus 0.67 ± 0.34 cm). There was also a non-significant difference in the long-axis diameter [49]. In the subgroup of small LNs with a short-axis diameter < 10 mm, neither the long-axis diameter nor the Solbiati index was significantly different between malignant and benign LNs [49].

In cases of axillary and inguinal LN metastases, the Solbiati index is often falsely negative because these often very large LNs can contain a great amount of fatty tissue [23]. The tumor proportion must be pronounced in order to cause a noticeable reduction in the Solbiati index. In these anatomical regions, the cortex width and asymmetry of the cortex are considered to be the better criteria [23,32].

### 2.7. Short/Long-Axis Ratio

Some authors determined the ratio between the LN short-axis and long-axis (S/L ratio) [1,31,33,35,48]. The S/L ratio is a mathematically inverse Solbiati index.

#### 2.7.1. What Is Normal (Reference Values)

In cervical LNs, the ratio was 0.3–0.7, depending on the localization. The optimum cut-off value of the S/L ratio was determined in different cervical regions: submental (0.5), submandibular (0.7), parotid (0.5), upper cervical (0.4), middle cervical (0.3), and posterior triangle (0.4) [31]. In the abdomen, the mean values ranged between 0.41 and 0.52 in healthy probands with no case exceeding a value of 0.69 [1].

#### 2.7.2. What Suggests Malignancy

In the study by Lyshchik et al. [35], 75% of metastatic cervical LNs and only 18% of benign cervical LNs had a short-to-long-axis diameter ratio above 0.5 (*p* < 0.01) [35] [Figure 8].

### 2.8. Influence of Age and Gender on Lymph Nodes Size and S/L-Axis Ratio

In a study involving sonographic examination of cervical LNs in 133 healthy subjects, the LNs in subjects aged 20 to 29 and 30 to 39 were often smaller than in probands aged 40 to 49 and 50 or older [25]. However, the differences were not statistically significant. Other characteristics, such as the shape and edge sharpness of the cervical LNs, did not vary significantly according to age or gender. A hyperechoic LN hilum was significantly more common in both genders with increasing age [25]. However, in the study of Osanai et al. [55], this did not affect the diameter of the short-axis, and no significant relationship between age and the S/L ratio was observed.

In the study of Okumuş et al. [48] involving healthy subjects, the S/L ratio of cervical, submental, and submandibular LNs of both sides of the neck did not differ between male and female healthy subjects and was not correlated with their age [48].

Ying et al. [56] compared cervical LN characteristics between 20 healthy Chinese and 20 white subjects and did not find any significant differences with regard to their number, size, distribution, and grayscale and vascular characteristics.

Overall, current evidence indicates that lymph node size and S/L-axis ratio are largely independent of age, gender, and ethnicity in healthy individuals.

**Statement 1.**  *Short-axis diameter is more relevant than long-axis.*

**Statement 2.**  *Cut-offs depend on anatomical region.*

**Statement 3.**  *Size alone is insufficient; always combine with shape, vascularity, and stiffness.*

### 2.9. Shape

#### 2.9.1. What Is Normal (Reference Values)

Normal LNs usually have smooth borders.

#### 2.9.2. What Suggests Malignancy

**Figure 9:**
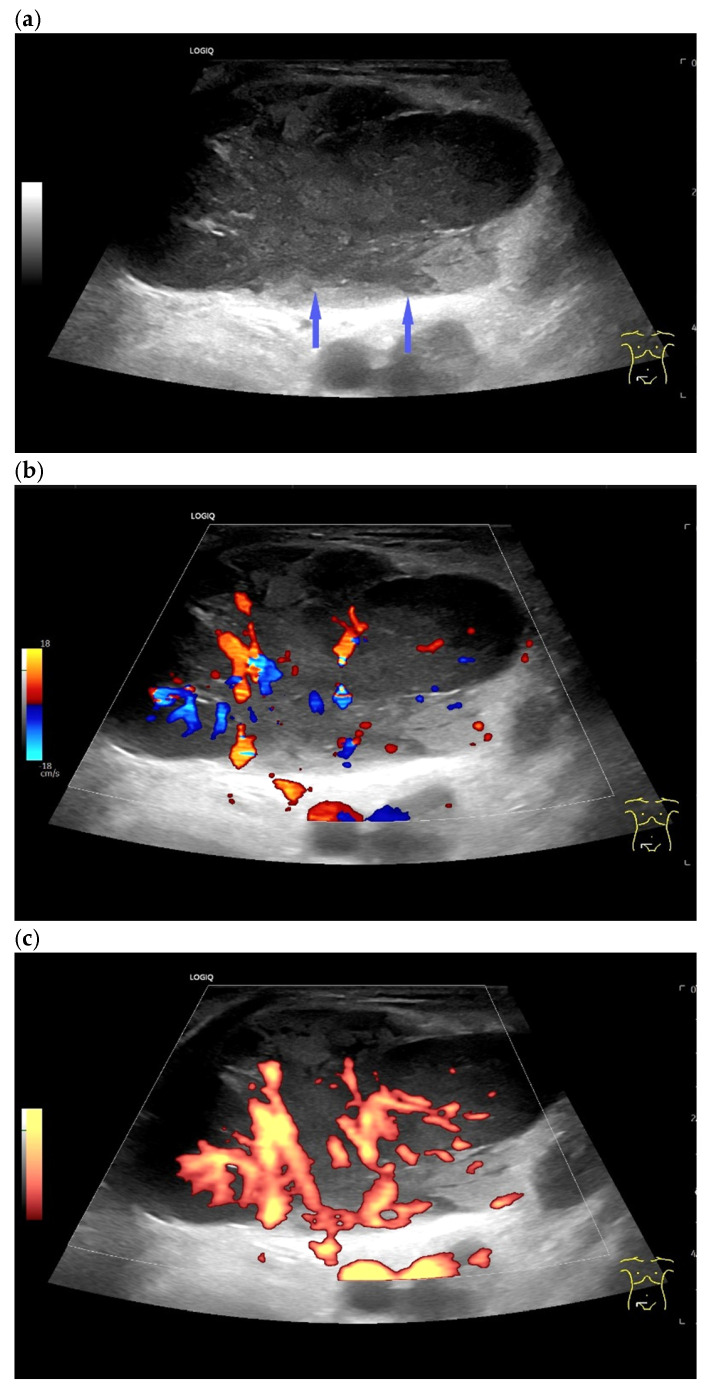
Diffuse large B-cell lymphoma. LN measuring 55 × 35 mm, rounded, very hypoechoic, inhomogeneous. Blurred border (arrows) (**a**). No central hilum can be identified in color Doppler imaging (**b**). Power Doppler shows two feeding vessels on the longitudinal side, which branch irregularly (**c**).

Metastatic tumors and malignant lymphomas, especially high-grade lymphomas, can exhibit growth beyond the capsule, which appears as indistinct borders on US [Figure 9].

#### 2.9.3. Overlap and Pitfalls

In cervical LN metastases from oral squamous cell carcinoma, malignancy was confirmed in 93% of the well-defined LNs and 63% of poorly defined LNs, respectively. A well-defined border is, therefore, very consistent with metastasis [39], but cannot be used to rule in malignancy. In malignant lymphomas, LNs can fuse together in large clusters and lose their demarcation (so-called “bulky lesions”).

### 2.10. Uniformity of Cortical Thickness

#### 2.10.1. What Is Normal (Reference Values)

Healthy LN has a uniform cortex.

#### 2.10.2. What Suggests Malignancy

**Figure 10:**
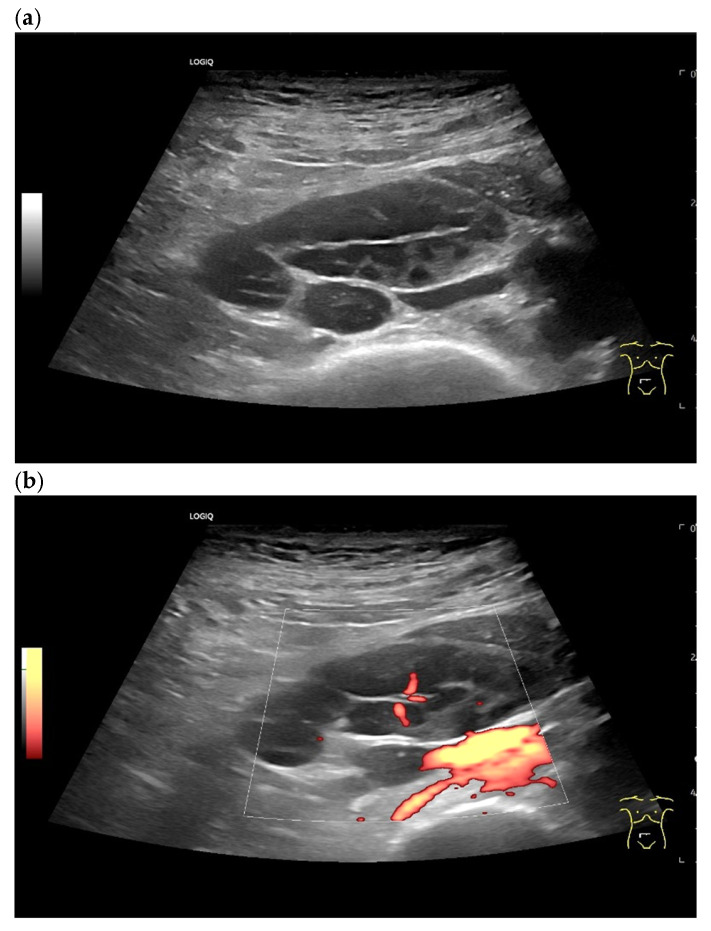
Chronic lymphocytic leukemia with watch-and-wait management. An enlarged paracaval LN measuring 30 × 20 mm is characterized by a compressed, narrow LN hilum and multiple hypoechoic lesions in the cortex (**a**). No feeding vessel is visible on power Doppler, but there are some small central vascular branches (vascular pattern II) (**b**).

**Figure 11:**
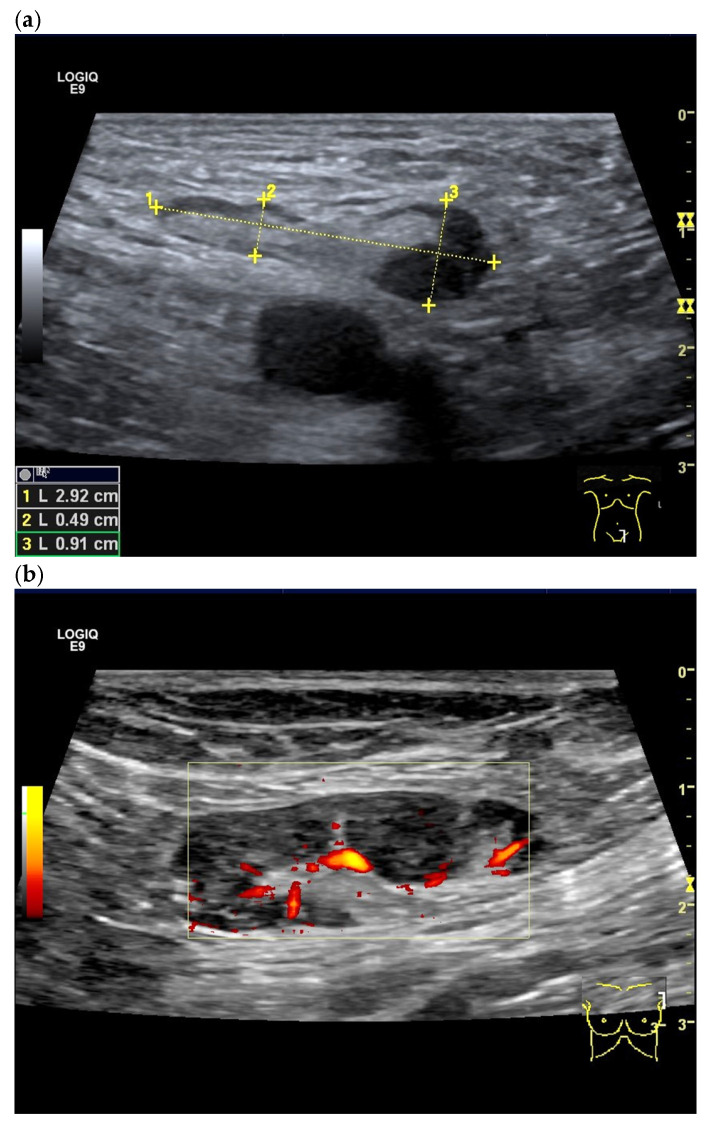
Inguinal LN with asymmetric cortical thickening. LN metastasis of gastric carcinoma (**a**). Enlarged painful axillary LN after vaccination in the left upper arm. Somewhat irregular cortex, with centrally located vessels (**b**). Spontaneous regression of findings in follow-up.

Asymmetric cortical thickening [26,53,57] and focal hypoechoic changes in the LN cortex [53,58] may both indicate focal malignant infiltration [Figure 10 and Figure 11].

#### 2.10.3. Overlap and Pitfalls

However, some regional LNs also exhibit varying cortical thickness. Otherwise, some tumors, especially lymphomas, exhibit uniform, diffuse cortical infiltration and thickening [Figure 8b].

### 2.11. Heterogeneity and Echogenicity

#### 2.11.1. What Is Normal (Reference Values)

Both the LN cortex and hilum normally have a homogeneous structure.

#### 2.11.2. What Suggests Malignancy

Tumor infiltration can lead to focal hypoechoic lesions [57] or inhomogeneity [33]. A necrosis can branch out within an LN with anechoic (liquid) components. Diffuse tumor infiltration leads to general or diffuse inhomogeneity, hypoechoic changes, and compression with potential obliteration of the LN hilum. Sometimes, LNs only become visible due to their pronounced hypoechoic echogenicity and rounded shape. Follicular lymphoma, in addition to its round shape and markedly hypoechoic echogenicity, can exhibit diffuse, finely spotted cortical lesions, reflecting the enlarged neoplastic follicles [58].

#### 2.11.3. Overlap and Pitfalls

Inflammatory processes or some infectious diseases can also lead to melting abscesses and necroses.

**Figure 12:**
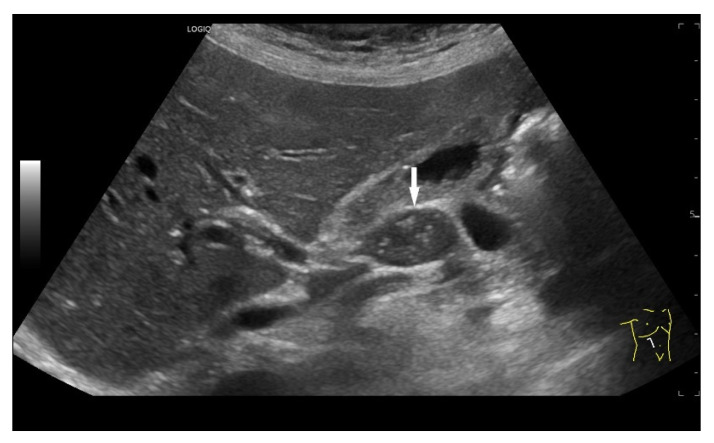
Sarcoidosis. Rounded oval LNs (with) small dot-like calcifications.

A homogeneous cortical pattern does not exclude malignancy, and malignant LNs can also appear homogeneous and hypoechoic. In a study of oral squamous cell carcinoma patients, 83% of LN metastases were homogeneous and 88% hypoechoic, respectively [39]. Due to deposits of thyroglobulin, cervical LN metastases of papillary thyroid carcinoma may be hyperechoic [59]. This particular appearance must be differentiated from the broad echogenic LN hilum, which is frequently observed in older patients. Calcifications in lymph nodes may indicate granulomatous inflammation [Figure 12] or malignancy [60].

B-mode US features like LN size, hypoechogenicity, presence of an echogenic hilum, and smooth borders are not reliable criteria for the differential diagnosis, especially of cervical LNs. In particular, in tuberculosis-endemic areas, the differential diagnosis between tuberculous and malignant LNs is difficult [61]. As in some malignant LNs, tuberculous LNs are predominantly hypoechoic with a heterogeneous pattern, intranodal necrosis, indistinct margins, and lymph node conglomerates. Areas of fusion of necrosis within the nodes can lead to abscesses [61].

**Statement 4.**  *Normal lymph nodes typically show a smooth, well-defined border and an elongated oval shape.*

**Statement 5.**  *Rounding of lymph nodes (increase in the short-axis relative to the long-axis) is associated with pathological involvement, including metastases and malignant lymphoma, but may also occur in reactive, granulomatous, or tuberculous lymphadenopathy.*

**Statement 6.**  *Well-defined borders do not exclude malignancy; metastatic lymph nodes may present with sharp margins, particularly in early stages.*

**Statement 7.**  *Indistinct or blurred borders suggest extracapsular extension and are more frequently seen in advanced metastatic disease or high-grade lymphomas.*

**Statement 8.**  *Confluent or bulky nodal masses with loss of individual node demarcation are characteristic of malignant lymphoma.*

**Statement 9.**  *Shape assessment alone is insufficient for reliable differentiation and must always be interpreted in combination with size, cortical morphology, vascular architecture, and stiffness.*

### 2.12. Color Doppler Imaging (CDI)

The vascular architecture/vascular pattern in color Doppler imaging (CDI), and the resistive index (RI) and pulsatility index (PI) are used to assess vascularity and arterial inflow resistance.

For Doppler examination of the LN hilar vessel, a low pulse repetition frequency and a low wall filter are selected. The color gain is used to select an intensity with a clear color signal in the vessel in the absence of blooming artifacts.

Sensitive Doppler techniques and microvascular imaging have improved the detailed vascularization of an LN’s blood supply [51,62,63,64,65,66,67,68].

#### 2.12.1. What Is Normal (Reference Values)

The blood supply to a normal LN is provided by a central artery in the hilum, which branches out into the periphery in a “tree-like” pattern. Drainage occurs via a similarly designed venous vessel. Inflammatory LNs are typically highly vascularized without altering the predominantly hilar vascular architecture. An exception is tuberculous LNs with melting abscesses.

#### 2.12.2. What Suggests Malignancy

Metastatic LNs exhibit varying degrees of vascular destruction. CDI criteria for malignancy and, in particular, metastatic infiltration include focal perfusion defects, subcapsular vessels, displacement or disappearance of intranodal vessels, and an aberrant vessel course [44]. The central LN hilum and its vessels may be destroyed with a loss of hilar vascular supply. Aberrant peripheral vessels are typical of metastatic LNs and represent tumor neovascularization. Vascularization may also be mixed with central and peripheral components [69]. The angioarchitecture of malignant lymphomas is highly variable. As with normal or reactive LNs, there may be a central vessel, but there may also be a vascular pattern similar to that seen in metastatic lymph nodes [70].

#### 2.12.3. Overlap and Pitfalls

In the study by Chen et al. [42], approximately one-third of the benign LNs corresponded to granulomatous inflammation or tuberculosis. Of these patients, only 53.1% had a central vascular hilum.

**Figure 13:**
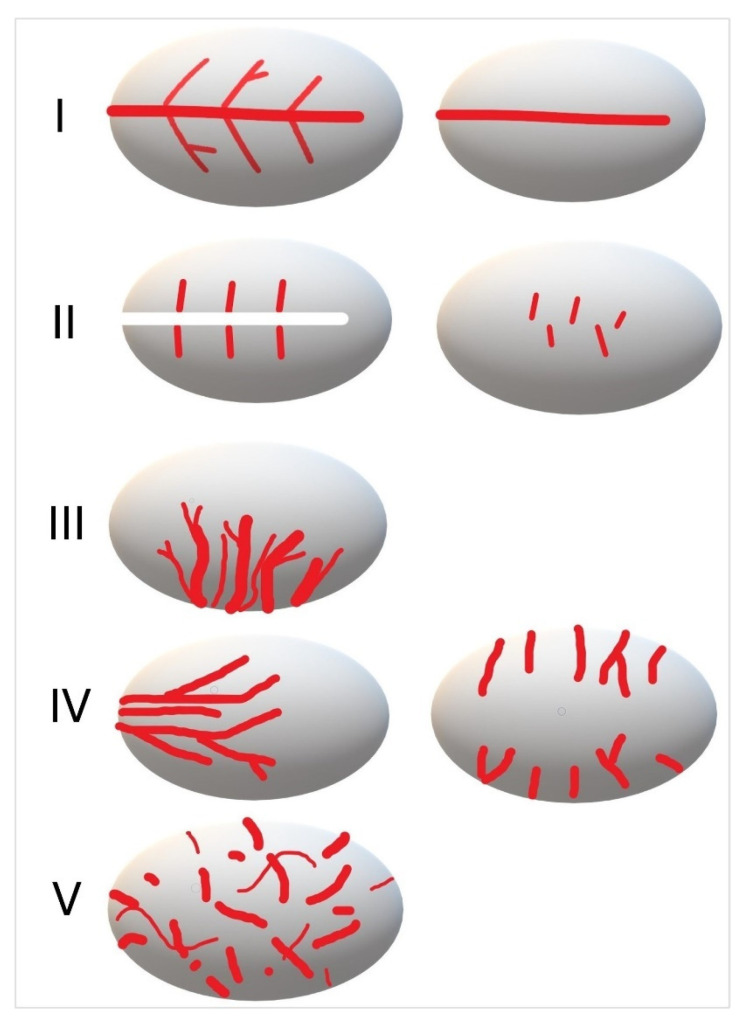
Vascular pattern of benign and malignant LNs: I—Central longitudinal vessels (with or without branches). II—Small central vessel segments, in the hilum area or central area. III (indeterminate pattern)—Several vessels, partially branched, enter the LN in a few rows from the longitudinal side. IV—peripheral accessory vessels. V—diffuse chaotic vessels, branching irregularly, with avascular areas.

**Figure 14:**
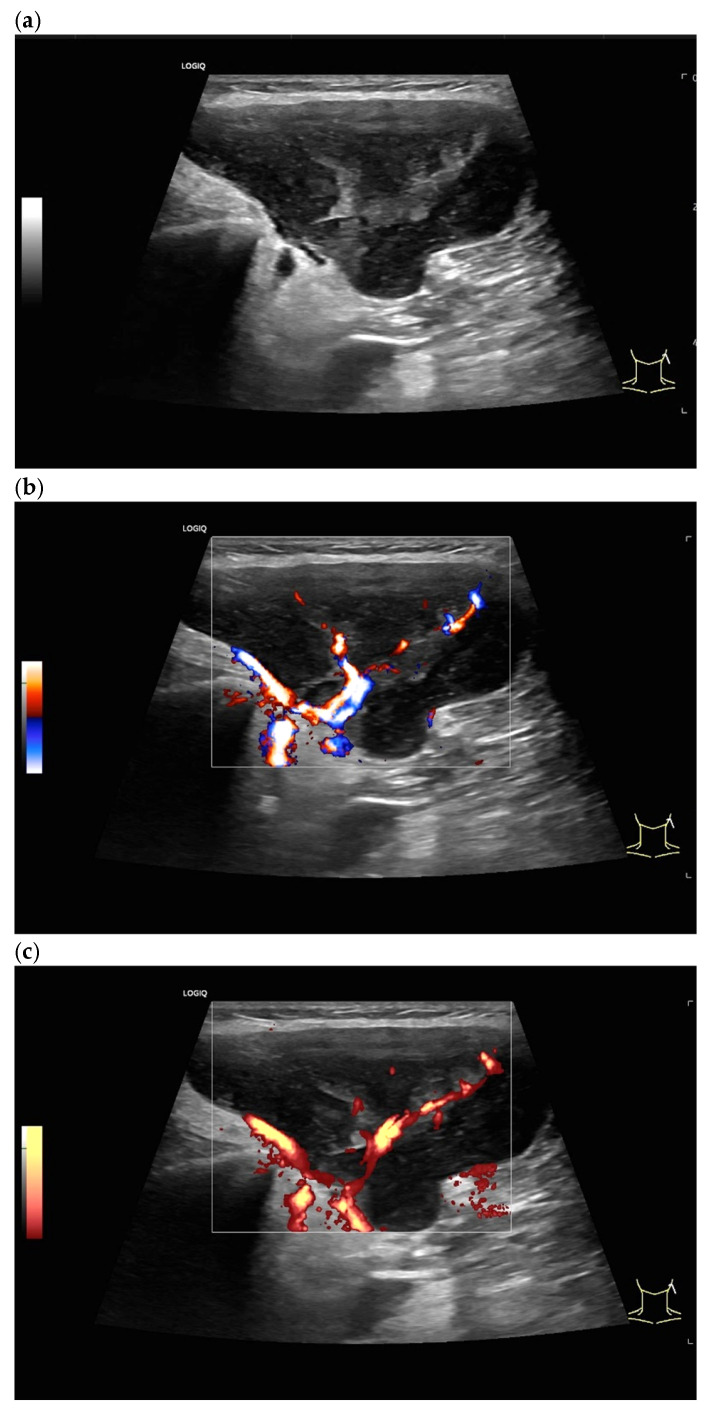
Inflammatory LN (50 × 31 mm) periauricular on the left side in parotitis. Heterogeneous echogenicity with echogenic internal structures (**a**). Central LN hilum with afferent and efferent vessels (**b**,**c**). Vascular pattern I.

**Figure 15:**
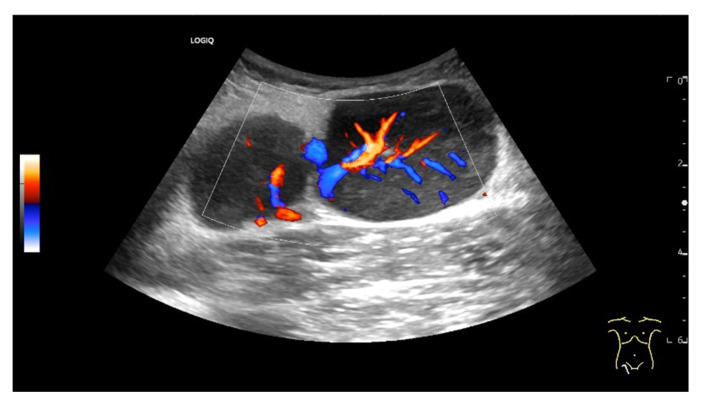
Hodgkin’s disease. Round, hypoechoic LN with central branching vessel, vascular pattern I.

**Figure 16:**
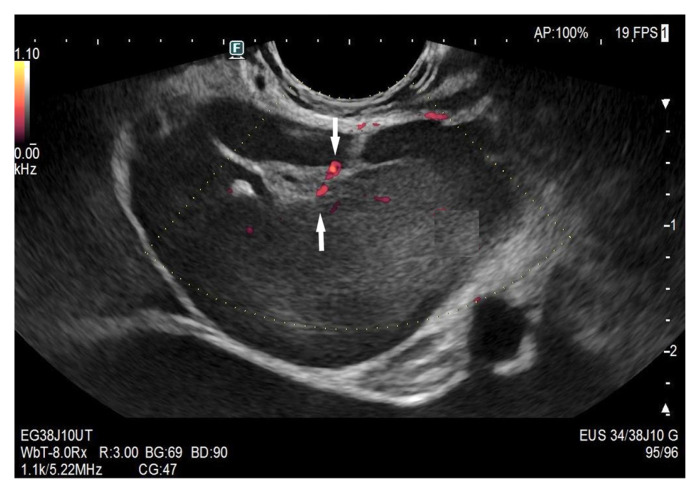
Enlarged mediastinal paraesophageal lymph node in EUS with rounded oval shape, visible echogenic lymph node hilum, absent central vessel, and branching central vessels (arrows), vascular pattern II. Intestinal tuberculosis with generalized lymphadenopathy.

**Figure 17:**
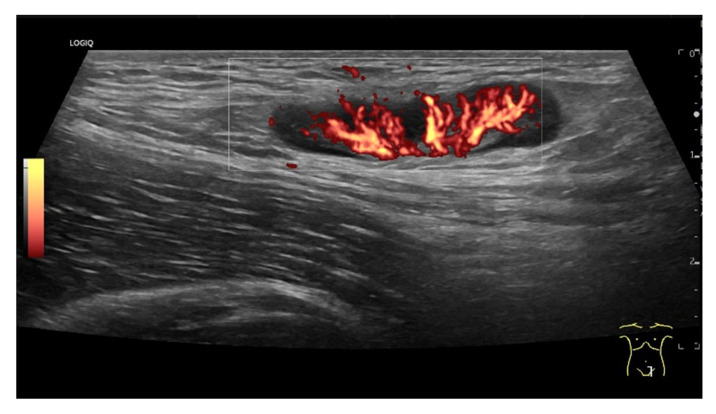
Inflammatory inguinal LN following urogenital infection. Significantly increased vascularization. Vessels supplying from the longitudinal side, vascular pattern III.

**Figure 18:**
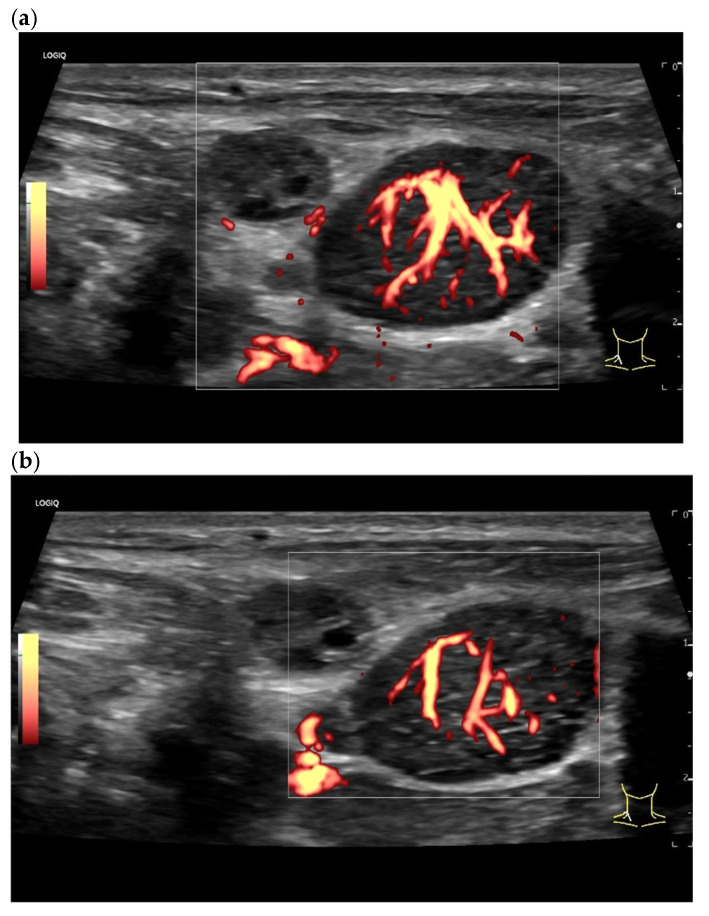
Mantle cell lymphoma. Round LN. Lateral feeding vessels with branches, vascular pattern IV (**a**,**b**).

**Figure 19:**
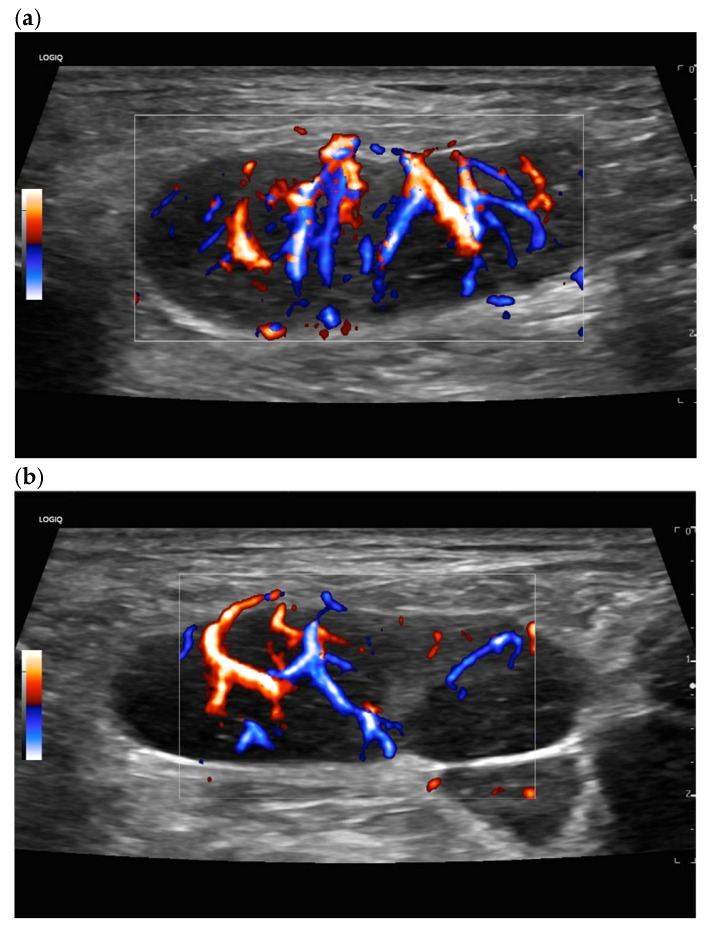
Chronic lymphocytic leukemia. Rounded oval LN with numerous peripheral lateral vessels and irregular branching (**a**,**b**).

**Figure 20:**
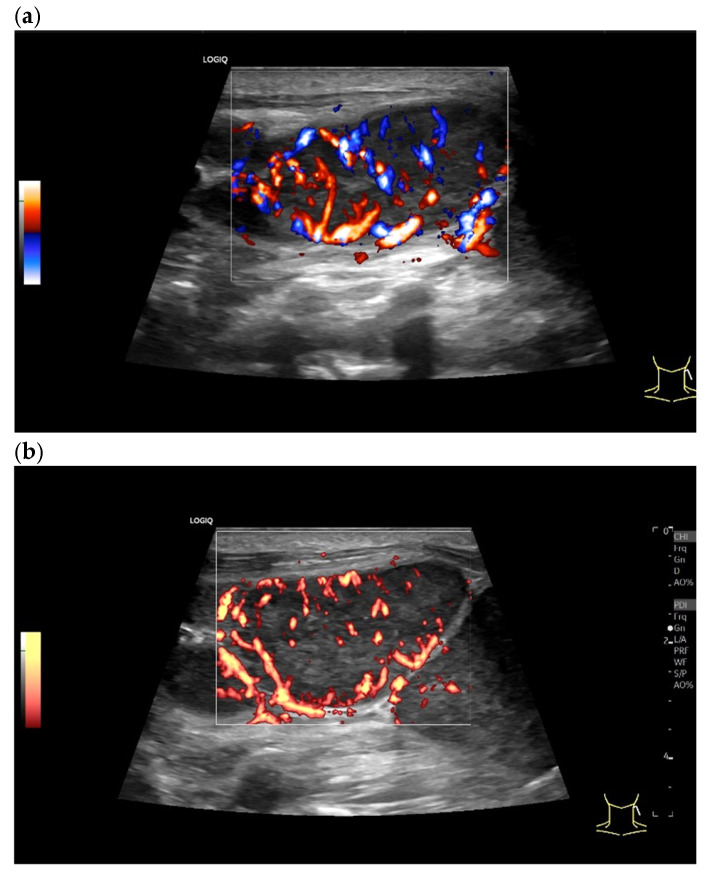
Hodgkin’s disease and acute Epstein–Barr virus infection. Rounded LN with inhomogeneous parenchyma. Peripheral vascularization and also diffuse central small vascular spots (vascular patterns IV and V; (**a**,**b**)).

**Figure 21:**
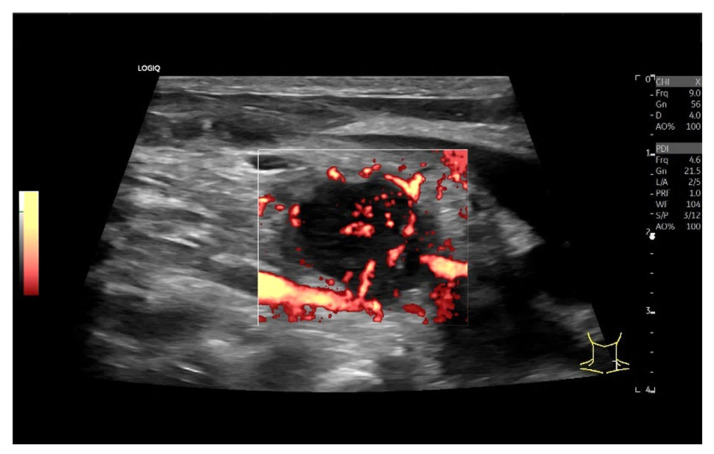
Malignant T-cell lymphoma. A central feeding artery is combined with abundant peripheral vessels and irregular diffuse vascular spots (vascular pattern V).

**Figure 22:**
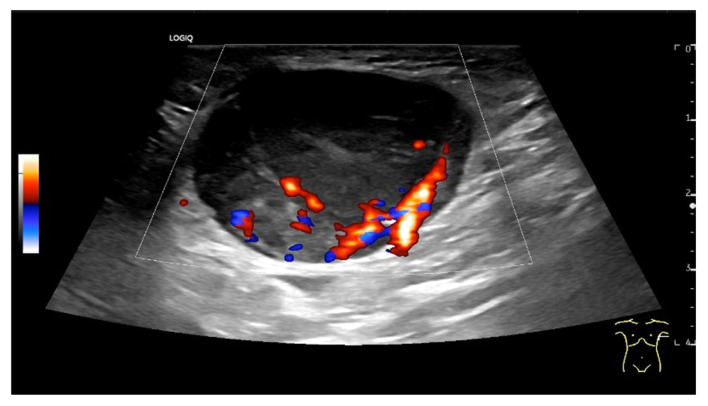
LN metastasis of a malignant melanoma with peripheral vessels and a large area without vascularization (vascular pattern IV).

According to Bialek et al., five vascular patterns are classified [70]: I—Longitudinal hilar vessel with symmetric or asymmetric branches or without branches. II—Short vessel segments distributed in the hilum or centrally, but not longitudinally in the hilum vessel. III—Multiple vessels, partially branching, entering the LN in a few rows from its longitudinal side. IV—Peripheral vessel distribution (short vessel segments or branching vessels). V—Irregular, chaotically branched vessels. Non-tumorous LNs usually exhibit vascular patterns I and II, metastatic LNs usually, but not always, III-V, and lymphomas can exhibit all vascular patterns. Type III is classified as indeterminate [70]. These types of vascular patterns, according to Tschammler and Bialek [44,70], are schematically outlined in Figure 13. Examples are presented in Figure 14, Figure 15, Figure 16, Figure 17, Figure 18, Figure 19, Figure 20, Figure 21 and Figure 22.

The visualization of the vessels depends on the use of the high-resolution transducer and the appropriate color Doppler imaging settings. Patient-related factors are less important in the case of superficial LNs.

### 2.13. Resistive Index (RI) and Pulsatility Index (PI)

In the afferent hilar vessel, systolic and end-diastolic velocities can be measured, and the resistive index (RI) and pulsatility index (PI) can be calculated. RI and PI are significantly lower in benign LNs compared to metastatic LNs. The increase in RI and PI reflects an elevated intranodal and thus also peripheral arterial pressure resulting from the space-occupying effect of metastatic infiltration within the LN capsule. If the LN vessels and, in particular, the hilum cannot be visualized, as is the case with small lymph nodes and very delicate vessels, for example, this measurement cannot be performed. In case of absent hilar vessels (for example, Type IV vascularization), velocities can be measured in peripheral vessels penetrating the LN’s capsule.

Different studies report similar cut-off values between benign and malignant LNs. Ghafoori et al. [33] report a cut-off for the RI of 0.69 (sensitivity 82% and specificity 81%). Ying et al. [71] report a cut-off for the RI of 0.7 (sensitivity 88%; specificity 78%), and Steinkamp et al. [52] report a cut-off for the RI of 0.8 (sensitivity 80%; specificity 94%).

For the PI, the cut-off values reported in studies were 1.35 (sensitivity 82%, specificity 68%) [33], 1.4 (sensitivity 82%, specificity 92%) [71], and 1,6 (sensitivity 94%, specificity 97%) [52].

#### 2.13.1. What Is Normal (Reference Values)

In general, RI values < 0.8 and PI values < 1.5 are used as cut-offs for benign LNs [72,73,74].

#### 2.13.2. What Suggests Malignancy

RI > 0.8 and PI >1.5 are considered to predict metastatic LNs [72,73,74].

Ying et al. [71] reported that a cut-off RI value of 0.8 enabled differentiation between LN metastases (RI > 0.8) and lymphomas (RI < 0.8) with an accuracy of 65% and 75%, respectively. Vascular displacement was helpful in distinguishing tuberculous LNs (accuracy: 67%) from reactive LNs and lymphomas (accuracy: 100% and 95%, respectively), while PI with a cut-off value of 1.5 helped distinguish between tuberculosis (PI < 1.5) and metastases (PI > 1.5) with an accuracy of 77% each. While the PI for tuberculous LNs was only slightly higher than in reactive LNs (1.3 versus 1.05), the PI values for lymphomas (1.95) and metastatic lymph nodes (2.2) were significantly higher [71].

#### 2.13.3. Overlap and Pitfalls

However, the RI and PI values in LN metastases, malignant lymphomas, and inflammatory lymphadenopathy overlap considerably, as not all LNs are evenly infiltrated [30].

In the study of Steinkamp et al., it was not possible to distinguish between lymphomas and metastases using RI and PI [52]. However, qualitative assessment of perfusion patterns was helpful in detecting malignant disease, as reactively enlarged LN exhibited greater hilar perfusion, while metastases exhibited increased peripheral perfusion. Lymphomas exhibited both increased central and peripheral vascularity.

**Figure 23:**
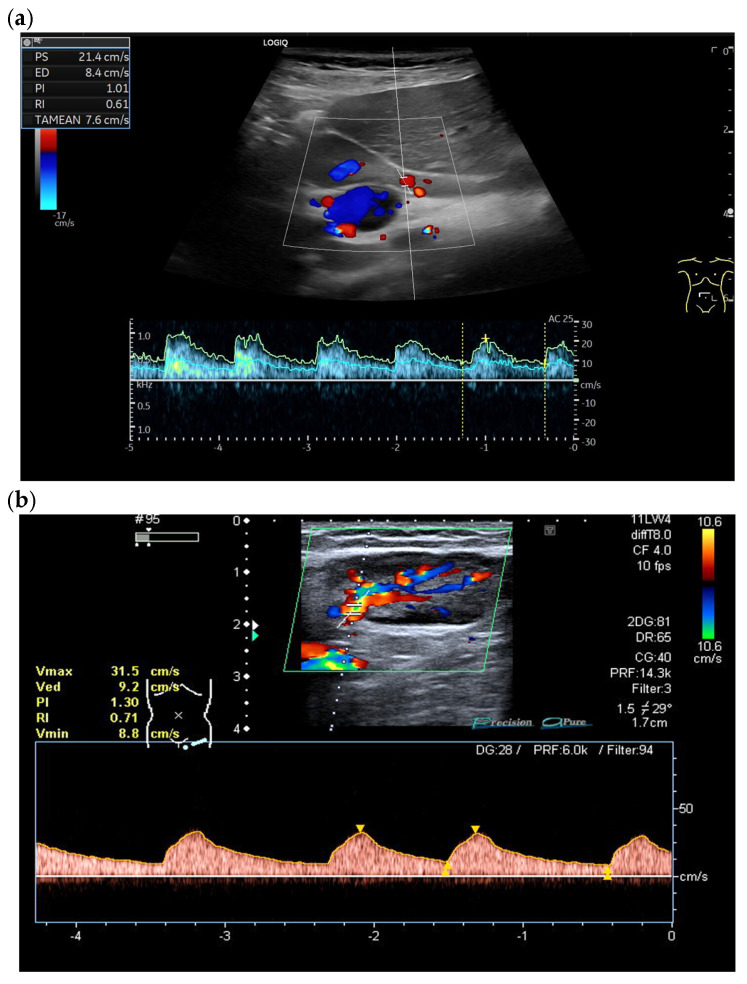

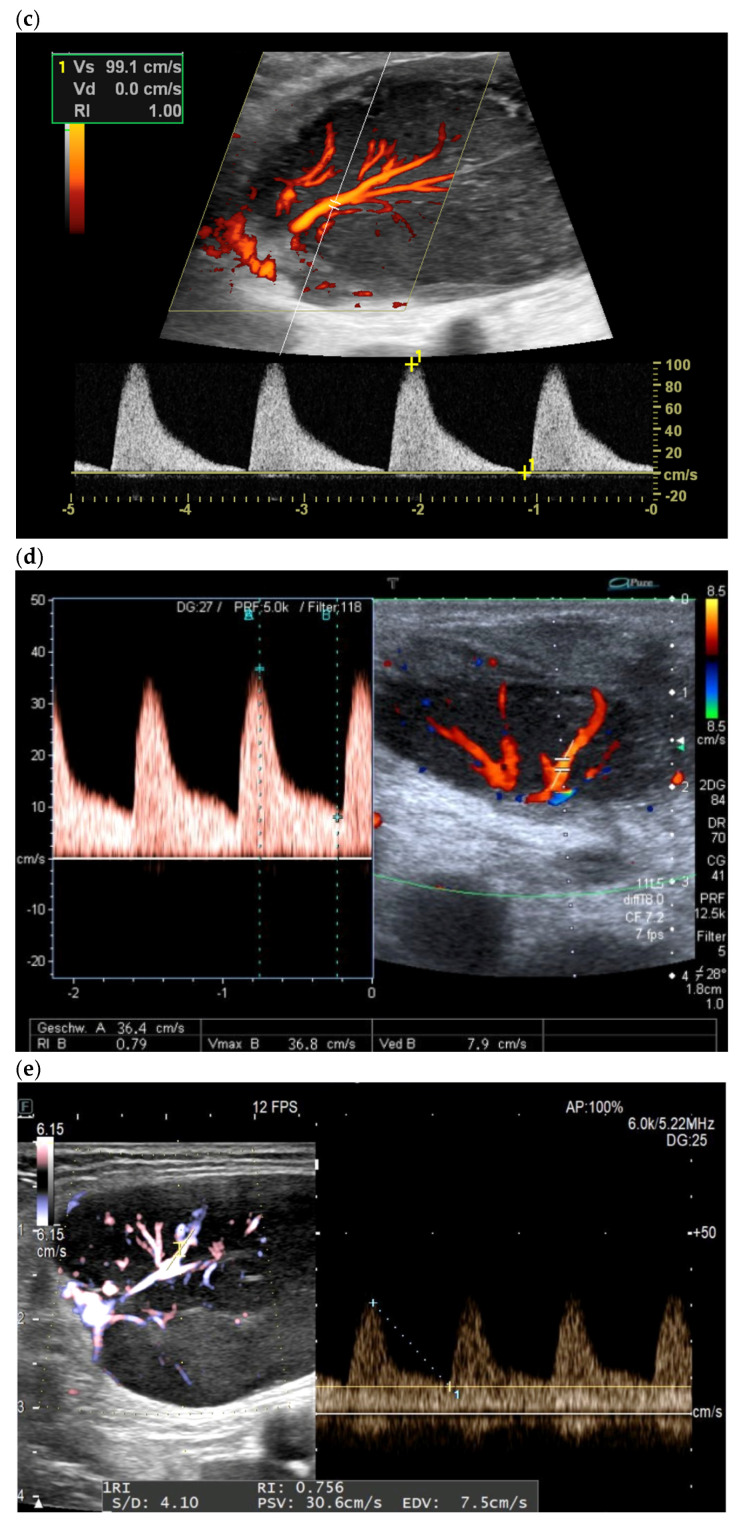
Measurement of RI < 0.8 and PI < 1.5 in a benign LN in the liver hilum (**a**) and RI of 0.71 in a patient with reactive lymphadenopathy in the inguinal region (**b**). In contrast, RI of 1.0 is indicative of malignant lymphadenopathy and cancer (**c**), whereas patients with malignant lymphoma show values in between (**d**,**e**). Follicular cell lymphoma (FCL) with RI 0.79 (**d**) and EBV-associated inguinal Hodgkin lymphoma with RI 0.76 (**e**).

It should be noted, however, that not every LN has a sufficiently strong hilar vessel from which a Doppler curve can be derived. Figure 23 demonstrates the measurement of RI and PI in a benign lymph node and the measurement of RI in several further LNs. Table 5 summarizes studies with cut-off values for RI and PI.

**Statement 10.**  *Normal and reactive LNs typically show a predominantly central (hilar) vascular pattern with regular, tree-like branching and preserved hilar architecture.*

**Statement 11.**  *Metastatic LNs frequently demonstrate peripheral, accessory, or chaotic vascularization, reflecting tumor-induced neovascularization and destruction or displacement of the hilar vessels.*

**Statement 12.**  *Malignant lymphomas exhibit highly variable vascular patterns, ranging from preserved central perfusion to mixed or diffuse peripheral vascularity; therefore, vascular pattern alone cannot reliably distinguish lymphoma from metastasis.*

**Statement 13.**  *Vascular pattern classification (types I–V) improves diagnostic stratification: patterns I–II are usually benign, patterns IV–V are strongly suspicious for malignancy, while pattern III remains indeterminate.*

**Statement 14.**  *The resistive index (RI) and the pulsatility index (PI) are generally lower in benign or inflammatory LNs and higher in metastatic LNs, but values show considerable overlap and are only interpretable if a suitable vessel can be measured.*

**Statement 15.**  *Absence of detectable vascularity does not exclude malignancy and may occur in small LNs, necrotic LNs, or with suboptimal Doppler sensitivity.*

**Statement 16.**  *Vascular assessment should always be integrated with B-mode morphology, elastography, and clinical context rather than used as a standalone criterion.*

### 2.14. CEUS

CEUS allows for the visualization of even the smallest vessels (e.g., microperfusion). Principles and recommendations are documented in the guidelines of the European Federation of Societies for Ultrasound in Medicine and Biology (EFSUMB) [75]. The statements refer to the intravenous administration of the intravascular ultrasound contrast agent (UCA) SonoVue^®^ (Bracco Suisse SA, Geneva, Switzerland). This is to be distinguished from intradermal UCA applications for the identification of sentinel lymph nodes in breast cancer [76,77]. If the CDI fails to distinguish between the central and peripheral vascular architecture, this can be better visualized using CEUS. Non-perfused LN parts can be clearly demarcated; however, this does not lead to a reliable classification of the tumor, as even inflamed LNs may contain necroses or melting abscesses. However, distinguishing necroses is helpful in US-guided sampling. It is helpful to obtain vital tissue for histology and not to biopsy necroses.

CEUS can improve the accuracy of LN assessment (see chapter “Multiparametric ultrasound”) [78,79].

#### 2.14.1. What Is Normal (Reference Values)

The vast majority of benign cervical LNs exhibit centrifugal perfusion and homogeneous contrast enhancement [80].

#### 2.14.2. What Suggests Malignancy

Characteristics of metastatic LNs include heterogeneous and centripetal contrast enhancement and peripheral and spotted vascularization [60]. Most malignant cervical LNs exhibit centripetal perfusion and heterogeneous enhancement [80].

#### 2.14.3. Overlap and Pitfalls

A total of 80.3% of metastatic LNs and 68.4% of lymphatic tuberculosis cases showed a centripetal perfusion pattern. The majority of lymphomas (76.5%) showed complete homogeneous contrast enhancement [81,82].

In the study by Cui et al. [43], dynamic contrast-enhanced ultrasound (DCEUS) [83] with time–intensity curve (TIC) analysis was helpful in differentiating tuberculous from metastatic LNs [43]. However, the significance of quantitative measurements in DCEUS cannot yet be conclusively assessed [78].

**Statement 17.**  *CEUS enables visualization of microvascular perfusion beyond the sensitivity of conventional Doppler techniques and allows accurate detection of non-perfused areas, such as necrosis or abscess formation.*

**Statement 18.**  *Benign LNs typically show homogeneous, centrifugal enhancement, whereas malignant LNs more often exhibit heterogeneous, centripetal enhancement with irregular or peripheral perfusion.*

**Statement 19.**  *Inflammatory and granulomatous LNs, particularly tuberculous LNs, may demonstrate enhancement patterns similar to malignancy, limiting specificity.*

**Statement 20.**  *Lymphomas frequently show homogeneous enhancement, which may overlap with benign patterns.*

**Statement 21.**  *Quantitative CEUS and time–intensity curve analysis show promise but are not yet sufficiently standardized for routine clinical use.*

**Statement 22.**  *CEUS is currently not recommended as a routine standalone method for LN characterization but may provide diagnostic benefit in selected cases, especially (a) when Doppler findings are inconclusive, or (b) for guidance of targeted biopsy to avoid necrotic tissue.*

### 2.15. Superb Microvascular Imaging (SMI)

A high-resolution Doppler technique is superb microvascular imaging (SMI; Canon Medical Systems, Otawara, Japan), which is marketed by various companies under different names. These techniques offer high resolution in the visualization of microvascular structures [51,62,84]. With US-guided SMI, it is possible to visualize even the smallest vascular structures at a precapillary level and their architecture. In addition, the method allows quantitative measurements of vessel density [51,62]. SMI enables even higher resolution imaging of small and slow-flowing vessels compared to power Doppler. This is achieved by using an advanced Doppler algorithm without the need for an intravenous contrast agent. However, the use of UCA can further improve the imaging of the smallest vascular structures.

SMI has both a color and a monochrome mode for vascular imaging. In color mode, both B-mode and color information are displayed. The monochrome mode removes background information and displays only the vascular information [84].

Several studies show that microvascular imaging is more sensitive in depicting the neovascularization of metastatic LNs [22,63,64,65,66,67,68,84]. In a study by Sim et al. [84] involving cervical LNs, the vascular patterns differed significantly between the groups with benign and metastatic LNs, tuberculous versus metastatic LNs, and inflammatory necrotizing LNs versus lymphomas in SMI compared to power Doppler (PDUS). This concerned the distribution of the feeding vessels, the number of internal vessels, and the appearance of the internal LN vessels. However, there was also a considerable overlap between the groups here. In benign LNs, central vascularity was the most common distribution pattern for both PDUS (38.8%) and SMI (55.3%). In malignant LNs, a large proportion of LNs showed a central (43.5%) or avascular (29.0%) distribution on PDUS. However, on SMI, many malignant LNs showed mixed (58.1%) or peripheral (24.2%) patterns. In SMI, malignant LNs had a significantly higher number of internal vessels compared to benign LNs. In SMI, more malignant LNs with eccentric vascularity were detected than with PDUS (88.7% vs. 46.8%). With regard to the internal LN vessels, eccentric shape was most common in both metastases and tuberculous lymphadenitis in both PDUS and SMI. Metastatic LNs had a significantly greater number of internal vessels than those with tuberculous lymphadenitis [84,85], and an avascular pattern appeared more frequently in tuberculosis [85]. The combination of PDUS and SMI is helpful in the classification of indeterminate LNs [86].

A more precise delineation of the small LN vessels may be helpful in the pre-test probability of lymph node classification.

**Figure 24:**
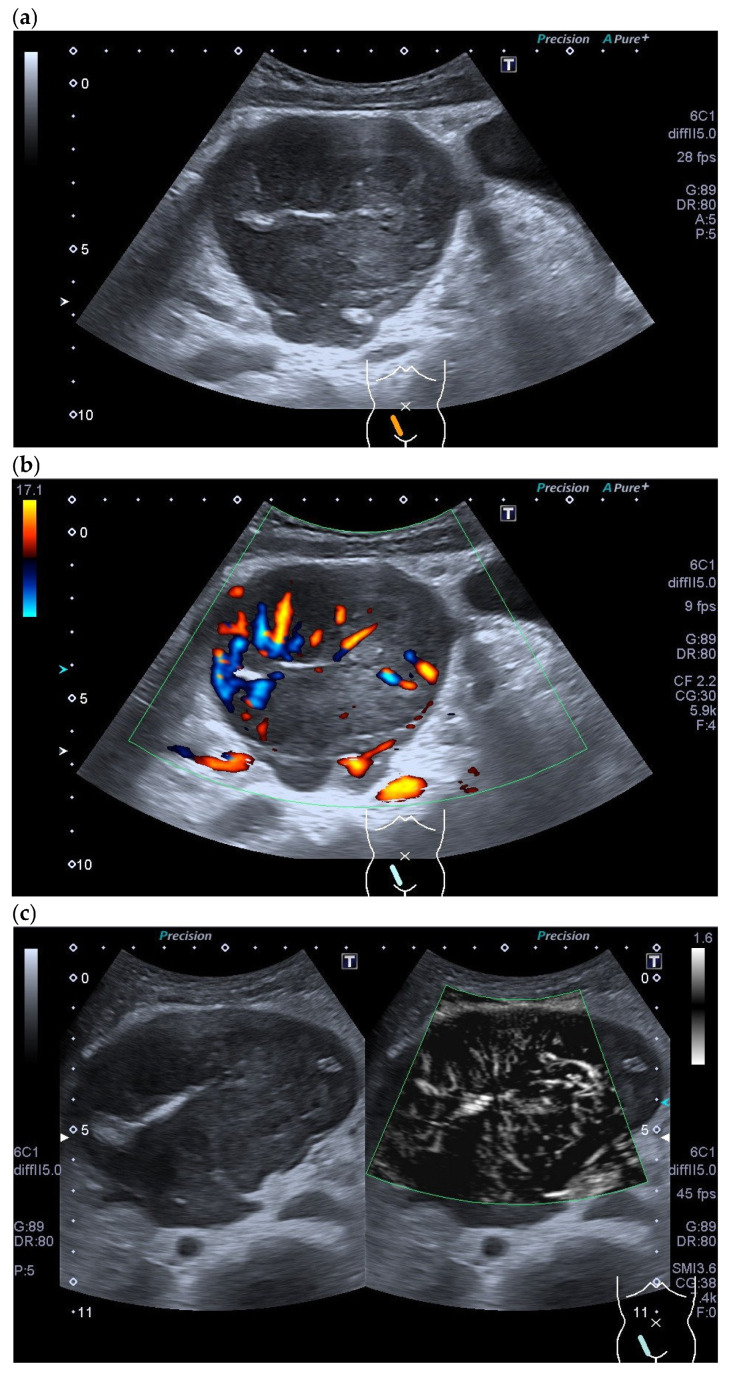

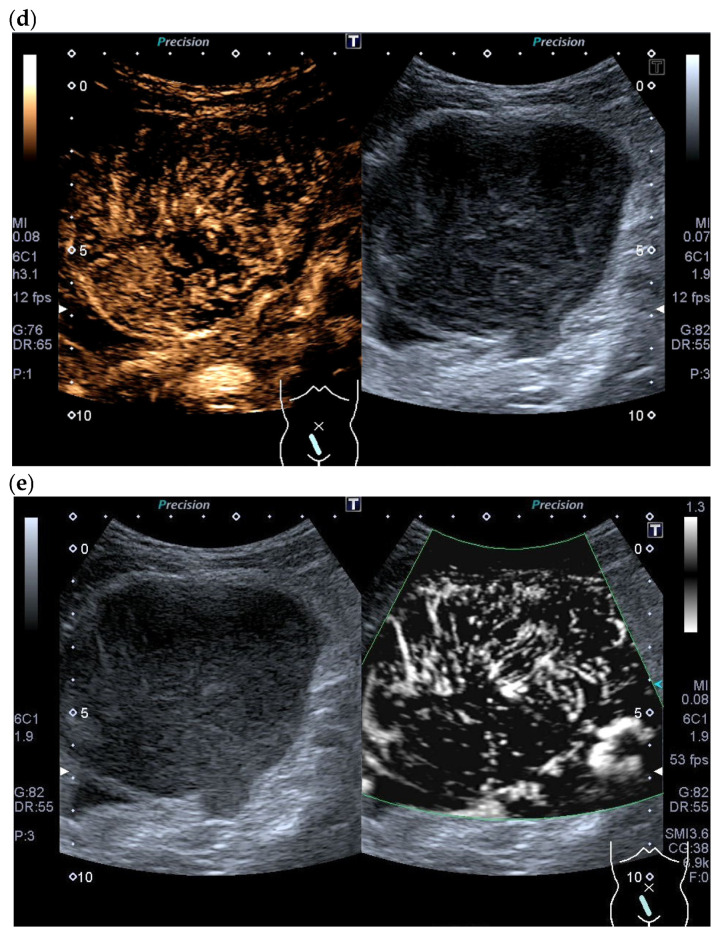
Non-Hodgkin lymphoma. Enlarged round hypoechoic LN in the right inguinal region with a compressed central lymph node hilum (**a**). No afferent central vessel is visible on CDI, but vessels branching off from the LN center, single peripheral accessory vessels (**b**). In non-enhanced monochromatic SMI (Canon Medical Systems, Otawara, Japan)), compared to CDI, a high density of numerous vessels branching from the center is visible (**c**). A central afferent vessel is also not distinguishable. In CEUS, centrally branching vessels, homogeneously enhanced areas, and non-enhanced areas are visible, thus representing a partially heterogeneous enhancement (**d**). In addition to the high density of central vessels and avascular areas, the CE monochromatic SMI also shows peripheral vessels (**e**).

An example of a lymphoma including CDI, CEUS, non-enhanced and enhanced SMI is presented in [Figure 24].

**Statement 23.**  *Non-CE or CE SMI can display finer vessels, which may influence the classification of some LNs in the vascular pattern.*

### 2.16. Elastography

The various elastographic techniques for imaging tissue elasticity of peripheral LNs can essentially be divided into two main groups: strain imaging and shear wave velocity measurement [69,73,87,88,89,90,91,92,93]. Principles and recommendations are documented in the guidelines of the professional associations [87,94,95,96,97]. In strain elastography, compression of a target tissue by a US probe produces a strain (displacement of one tissue structure by another) that is inversely proportional to tissue stiffness. The strain is lower in stiffer tissues than in softer ones. Malignant tissue exhibits increased stiffness compared to benign or normal tissue and therefore deforms less upon compression [73]. The EFSUMB guidelines for non-hepatic elastography applications recommend elastography for superficial and mediastinal LNs as a complementary method in distinguishing between benign and malignant LNs. In addition, strain elastography is helpful in selecting a suitable LN for US-guided or EUS-guided sampling. The same applies to the detection of focal lesions of suspected malignancy in the LN for targeted needle biopsy [87].

**Figure 25:**
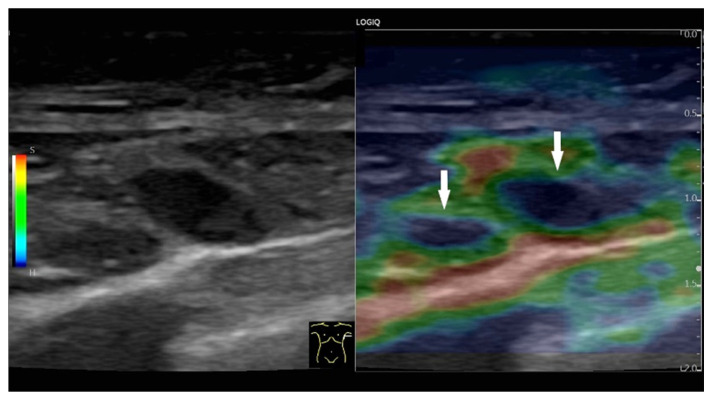
Hypoechoic heterogeneous axillary LNs (arrows), Level I on the side of breast carcinoma. Strain elastography shows the lymph nodes to be predominantly hard. Only a narrow green border is visible at the periphery.

A large number of studies have been conducted under the assumption that tumor tissue in LNs is stiffer compared to physiological or reactively activated LN tissue [42,49,98,99,100,101] [Figure 25].

### 2.17. Strain Elastography

In normal LNs, the cortex is significantly stiffer than the hilum [73]. Qualitative stiffness scores (from 1 to 4 or 1 to 5, correlating with the percentage of blue = stiff area) and the semi-quantification of stiffness differences between the LN and its surrounding using the strain ratio (SR) are used for differentiation [46]. Meta-analyses detected satisfactory accuracy for both qualitative strain scoring and SR, with advantages for the semi-quantitative approach. Ying et al. [102] calculated pooled sensitivities of 74% (specificity 90%) and 88% (specificity 81%) for qualitative real-time elastography and SR, respectively. Ghajarzadeh et al. [103] calculated very similar pooled sensitivities of 76% (specificity 80%) and 83% (specificity 84%) for the scoring system and the SR [103].

When using the strain elastography color scale, it is important to pay attention to the color scheme. Ultrasound manufacturers in Europe and the USA indicate the tissue property “hard” with the color “red” in their elastography modules, while Japanese companies use the color “blue” for “hardness” [104]. However, the color scheme can be preselected and customized on current ultrasound devices.

#### 2.17.1. What Is Normal (Reference Values)

Predominance of soft tissue (>50% of nodal area) and a strain ratio < 1.5 compared to surrounding tissue are suggestive of benignity.

#### 2.17.2. What Suggests Malignancy

In general, stiffness of more than 50% of the nodal area and an SR between LNs and surrounding tissue > 1.5 are considered to indicate malignancy [35,73].

#### 2.17.3. Overlap and Pitfalls

The subjectivity of the scoring systems, the usage of different scoring systems, the varying experience of the investigators, and the heterogeneity of included studies were considered limiting factors [103].

### 2.18. Shear Wave Elastography

Since shear waves propagate faster in stiff tissue, shear wave velocity (SWV) is proportional to tissue stiffness and can, therefore, be used as a surrogate marker for stiffness. The US systems show the velocity (m/s) and/or the maximum shear elastic modulus (kPa).

A meta-analysis of shear wave elastography (SWE) of superficial LNs showed a pooled sensitivity of 81% and 85% for the detection of malignancy, but data heterogeneity was considerable [100]. The included studies used acoustic radiation force impulse imaging (ARFI) and supersonic shear imaging (SSI). Sensitivity and specificity did not differ between ARFI and SSI. The authors considered SWE to be a useful additional examination. However, the various cut-off values for LN metastases of different primary tumors varied: 1.9–3.3 m/s and 19–57 kPa [100].

Neither the meta-analysis nor the guidelines define cut-off values for the differential diagnosis between malignant and benign LNs, nor do they specify cut-off values for different pathologies. Because SWE measurements vary substantially between manufacturers and acquisition protocols, a universally applicable cut-off value for LN stiffness cannot be defined.

Kanaragaju et al. [105] examined benign and malignant cervical LNs using strain elastography and SWE. On strain elastography, the majority of benign LNs demonstrated elastography patterns 1 and 2, and the majority of malignant LNs had patterns 3–5. Sensitivity, specificity, and accuracy of SE were 94.1%, 93.9%, and 94%, respectively [105]. The mean SWV of benign LNs (1.670 ± 0.367 m/s) differed significantly from malignant LNs (2.965 ± 0.826 m/s; *p* = 0.000). The cut-off value was 2.05 m/s with a sensitivity of 88.2% and a specificity of 84.8% [105].

Lerchbaumer et al. [49] performed elastography on benign and malignant cervical LNs (of various origins) using SWE. All malignant LNs exhibited significantly higher stiffness than benign LNs (SWE velocity 2.63 ± 1.03 m/s versus 1.72 ± 0.69 m/s) [49]. In the subgroup of malignant LNs with a Solbiati index > 2 and only significantly different short-axis diameters, the stiffness was again significantly higher in the malignant LNs (SWE velocity 2.45 ± 0.65 m/s versus 1.69 ± 0.57 m/s) [49]. In the subgroup of LNs with a short-axis diameter < 9 mm, only SWE showed significant differences (SWE velocity 2.27 ± 0.88 m/s versus 1.69 ± 0.7 m/s), not the long and short-axis diameters or the Solbiati index [49]. The higher stiffness observed in small malignant lymph nodes on SWE may reflect increased cellularity and desmoplastic changes due to early tumor infiltration, which means replacement of lymphoid tissue by tumor cells [41].

In the study of Chen et al. [42] with LNs in the cervical, axillary, and inguinal regions, peripheral malignant LNs exhibited a significantly higher stiffness (Young’s modulus values) on SWE than benign ones (49.38 ± 29.96 kPa vs. 25.00 ± 14.42 kPa, *p* < 0.001). For Young’s modulus values > 25.46 kPa, the AUC, sensitivity, specificity, PPV, NPV, and accuracy for identifying malignant LNs were 0.807, 0.787, 0.750, 0.926, 0.471, and 0.780, respectively. To distinguish benign LNs from lymphomas, the cut-off value was 25.03 kPa, with AUC, sensitivity, specificity, PPV, NPV, and accuracy of 0.727, 0.754, 0.719, 0.827, 0.622, and 0.742, respectively. To distinguish between benign and metastatic LNs, a cut-off value of 36.97 kPa resulted in AUC, sensitivity, specificity, PPV, NPV, and accuracy of 0.872, 0.757, 0.875, 0.930, 0.622, and 0.794, respectively [42]. In this study, there was no significant correlation between long or short-axis and SWE results in any of the groups [42].

In the study of Lahtinen et al. [106], using a 3 mm ROI window in the cortex of inguinal LNs, the benign LNs had a lower mean Young’s modulus value (7.68 ± 3.82 kPa; range, 3.41–15.40 kPa) compared to the malignant LNs (15.81 ± 10.61 kPa; range, 3.86–36.45 kPa) [106].

**Statement 24.**  *Malignant LNs are generally stiffer than benign or reactive LNs, reflecting increased cellularity, fibrosis, and tumor infiltration.*

**Statement 25.**  *Strain elastography allows qualitative and semi-quantitative assessment of stiffness; predominance of stiff tissue (>50% of LN area) and a strain ratio > 1.5 compared to surrounding tissue are suggestive of malignancy.*

**Statement 26.**  *Shear wave elastography (SWE) provides quantitative stiffness measurements and can detect malignancy even in small LNs with inconspicuous B-mode features, but reported cut-off values vary widely between studies and ultrasound systems.*

**Statement 27.**  *Normal LNs show relative softness, with the cortex being stiffer than the hilum; this internal stiffness gradient may be lost in malignant infiltration.*

**Statement 28.**  *Considerable overlap exists between benign, inflammatory, and malignant LNs, particularly in granulomatous disease and lymphoma.*

**Statement 29.**  *Elastography should be used as a complementary technique, increasing diagnostic confidence and aiding target selection for biopsy, rather than as a standalone discriminator.*

**Statement 30.**  *Routine SWE is currently recommended only in experienced centers, as standardized cut-off values and guideline-based thresholds are lacking.*

## 3. Multiparametric US

Although B-mode US, color Doppler imaging, elastography, and CEUS have criteria for benignity and malignancy, no single technique can reliably differentiate each LN.

Wakonig et al. [79] investigated the performance of these techniques individually and in combination for the differentiation of cervical LNs. B-mode US (homogeneity, LN shape and borders) had a sensitivity of 87%, specificity of 46%, PPV of 74%, NPV of 67%, and accuracy of 72% in differentiating malignant from benign cervical LNs [79].

CDI parameters (e.g., central, peripheral, or no hypervascularization) demonstrated a sensitivity of 85%, specificity of 67%, PPV of 82%, NPV of 72%, and accuracy of 79%. RI and PI were not calculated [79]. SWE (color-coded-SWE-map) performed a sensitivity of 71%, specificity of 90%, PPV of 92%, NPV of 64%, and accuracy of 78% [79]. The contrast enhancement pattern on CEUS and distribution homogeneity were as follows: centrifugal or centripetal pattern, general hypo- or hyperenhancement, and presence of avascular areas, which were rated as necroses, demonstrated a differentiation between benign and malignant cervical LNs with a sensitivity of 93%, specificity of 85%, PPV of 91%, NPV of 87%, and accuracy of 90% [79]. The combination of B-mode US and CDI resulted in 87% sensitivity, 44% specificity, 73% PPV, 65% NPV, and an accuracy of 73% [79]. The combination of SWE and CEUS showed a sensitivity of 91%, specificity of 77%, PPV of 87%, NPV of 83%, and accuracy of 90% [79]. The combination of B-mode US, CDI, and SWE resulted in a sensitivity of 96%, specificity of 77%, with 72% PPV and 82% NPV, and accuracy of 76% [79]. The combination of all four parameters (B-mode US, CDI, SWE, and CEUS) revealed the highest sensitivity of 97% at the cost of a very low specificity of 36%. The PPV was only moderate (73%), the NPV was 88%, and the accuracy was 83% [79]. In this study, CEUS alone or the combination of SWE with CEUS had the highest accuracy of 90% [79].

In a meta-analysis of five studies, Spiesecke et al. [78] found that the combination of non-enhanced US (B-mode US and CDI assessment of vascularity) and CEUS increased diagnostic certainty in the characterization of cervical LNs of unclear significance. The pooled sensitivity and specificity in the characterization of a malignant cervical LN using US were 76% (95% CI 66–83) and 80% (95% CI 45–95), respectively, compared to 92% (95% CI 89–95) and 91% (95% CI 87–94) for the combination of US and CEUS [78].

## 4. Lymph Node Biopsy, US-Guided Sampling

Histological confirmation is necessary for indifferent and, in particular, tumor-suspicious LNs. If the LNs are located superficially, but also in larger abdominal and retroperitoneal LNs, US-guided sampling is feasible. The principles and implementation are described in the guidelines of the European Federation of Societies for Ultrasound in Medicine and Biology (EFSUMB) on interventional US [107,108]. Abdominal and retroperitoneal LNs are visible to varying degrees in the US, depending on the patient’s constitution, their size, and their location in depth. Targeted compression can improve sonographic visibility and facilitate access between intermediate structures (intestines, vessels). With needle diameters of 18–16 G, tissue cores can be obtained that are sufficient for histology and immunohistology. Core needle biopsy provides information about LN architecture, and the histological material is also sufficient for immunophenotyping, molecular genetics, and molecular biology [109,110]. The authors affirm that US core needle biopsy with larger needle size (14–16 G) and increased sampling offers high efficiency (strong specificity, minimal complications) as a frontline approach, especially for lymphoma subclassification [109,110].

LNs in the mediastinal, perigastric, and periduodenal LN stations are often accessible for EUS-guided sampling, whereas pretracheal and parabronchial lymph nodes can be targeted by EBUS. We refer to the relevant literature [111,112]. In a retrospective study involving more than 6000 patients with cervical lymphadenopathy, adequate material was obtained in 92.19% of cases using US-guided sampling for differentiation of malignant vs. benign LNs. The overall accuracy was 91.7%. In cases with adequate material, sensitivity, specificity, and accuracy were 99.7%, 100%, and 99.46%, respectively [113]. A diagnosis of various types of lymphomas was possible in 80% of cases using core needle biopsy with or without immunohistochemistry [114].

**Figure 26:**
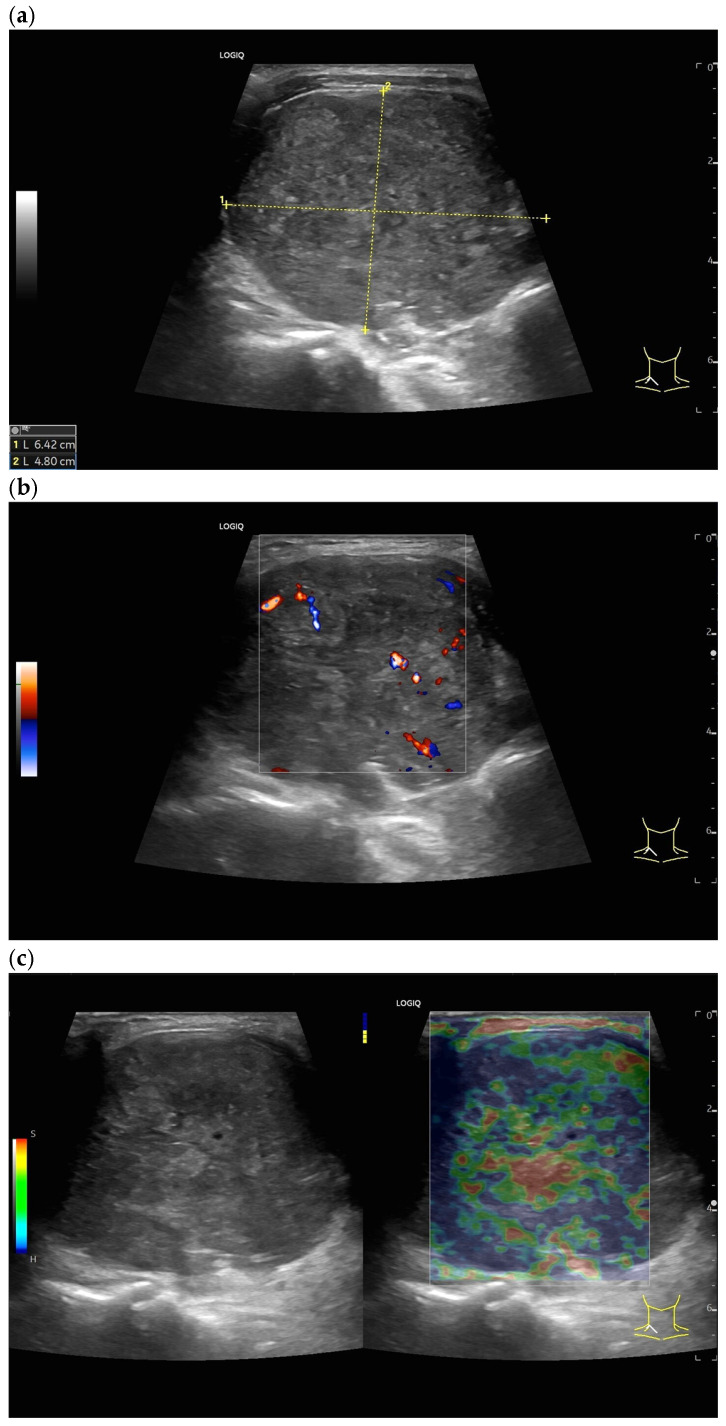

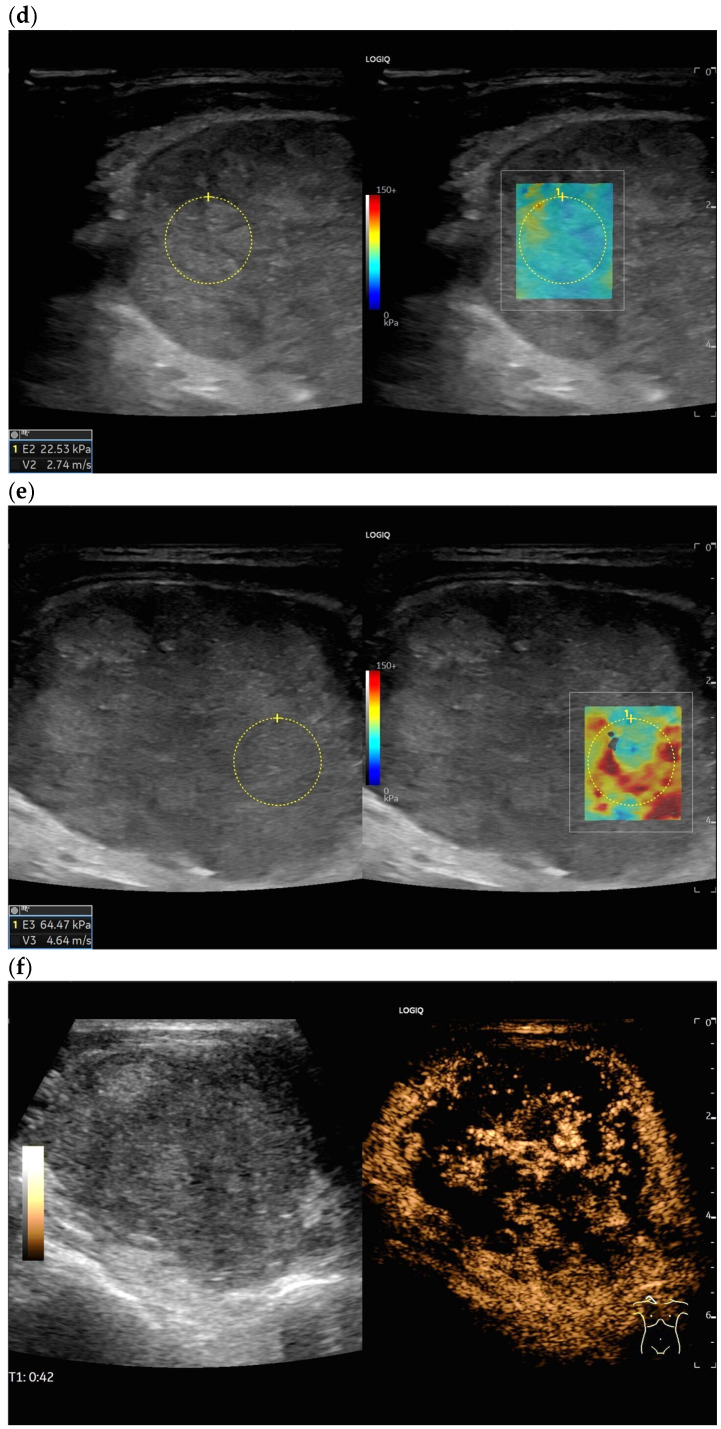
Cervical LN metastasis in a previously metastatic and treated colon carcinoma. US-guided sampling was used to obtain additional histologically evaluable material for further molecular pathology. The US appearance of the LN with a round shape, size (64 × 48 mm), heterogeneity, and diffuse vascular pixels in power Doppler are characteristic of malignancy (**a**,**b**). Strain elastography reveals stiffer areas but also “softer”/red-colored areas (**c**). Given the size of the metastatic LN, necrosis is likely. However, B-mode imaging does not allow conclusions to be drawn regarding the location of the necroses. SWE shows significantly different values measured at different locations, without these areas being distinguishable in B-mode imaging (**d**,**e**). CEUS using 2.0 mL SonoVue and a 9 MHz linear transducer showed that the lymph node was not enhanced in many areas, with some enhanced tissue islands (**f**). With knowledge of this pattern of findings, CEUS-guided targeted US-guided sampling of the enhancing lymph node areas was performed.

The most common, but rare, complication is post-interventional bleeding, which has been reported in 0.37% of cases [115]. Figure 26 shows a multiparametric LN assessment, in this case, prior to US-guided sampling.

Advances in needle technology combined with high-resolution US techniques have dramatically improved the diagnostic efficiency of US-guided core needle biopsy for LNs, resulting in surgical excision being required only very rarely for advanced histopathological workup or subclassification.

## 5. Future Development, Structured Diagnosis

**Figure 27:**
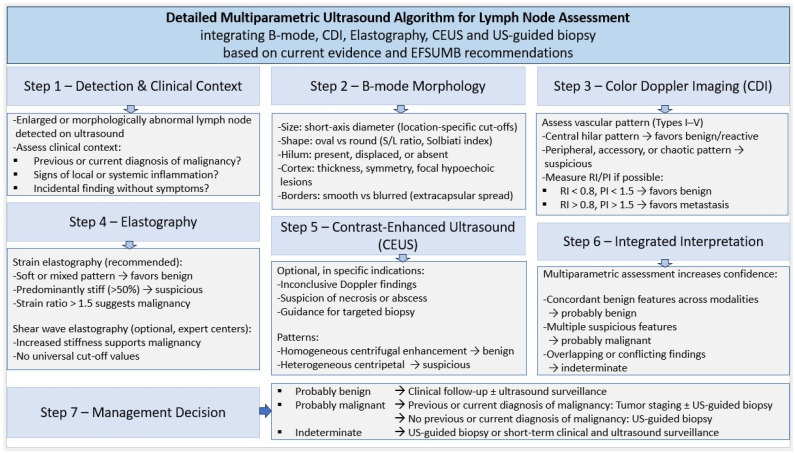
Detailed algorithm for lymph node diagnostics.

For MRI and CT, the likelihood of malignancy in LNs is assessed by a structured approach using the Node Reporting and Data System 1.0 (Node-RADS). It systematically classifies the degree of suspicion of lymph node involvement based on a synthesis of the assessment categories “size” and “configuration”. Assessment categories between 1 (“very low probability”) and 5 (“very high probability”) are defined, and probabilities of malignancy for different lymph node locations are derived from these and applied to different tumor entities [116,117,118]. The simple criteria of size and configuration are, in principle, applicable to the US. Potentially, the development of a US Node-RADS score could also include other US-detectable characteristics, such as strain elastography and vascular pattern, considering the patients’ medical history data. It also makes sense to develop an appropriate training curriculum that incorporates B-mode criteria, elastography, and CDI in a structured approach. A detailed algorithm for multiparametric lymph node diagnostics is shown in Figure 27.

While we routinely recommend strain elastography for indifferent or suspicious lymph nodes, SWE should currently be reserved for experienced centers, as there are no precise cut-off values or guideline recommendations. We would not routinely recommend CEUS, but rather in special situations based on the examiner’s indication, if a diagnostic gain is to be expected. In addition, we would use CEUS in the context of an ultrasound-guided biopsy for LNs where necroses or melting abscesses are to be expected and should be distinguished from vital tissue.

## 6. Conclusions

All LN entities, whether healthy, inflammatory, granulomatous, metastatic, or lymphoma, have typical characteristics on US [Table 6]. Due to special features resulting from the anatomical region, the specific type of inflammation, the grade of LN infiltration, the characteristics of the primary tumor of metastatic LNs, and also the specific type of lymphoma, the assignment of a particular LN based on these criteria is not unequivocal. Some inflammatory LNs, such as granulomatous inflammations (sarcoidosis, tuberculosis), can resemble malignant LNs. Also, malignant lymphoma shares some features with inflammatory/reactive LNs.

Multiparametric US, integrating B-mode, CDI, CEUS, and elastography, provides the most reliable non-invasive etiological assessment of an enlarged or otherwise morphologically abnormal LN.

The typical regional benign LN is no larger than 5 mm or 8 mm (depending on localization) in the short-axis, has an oval shape with a Solbiati index > 2 (corresponding to a S/L ratio < 0.5), a central echogenic lymph node hilum, and a uniformly thin and hypoechoic cortex. The hilar artery has a regular branching and a low-resistance Doppler spectrum (RI < 0.8; PI < 1.5). On strain elastography, a benign LN is not stiffer than the surrounding tissue. If LNs are abnormal in B-mode (enlarged, widened short-axis with round shape, hypoechoic, focal lesions), the type of vascularization (central, peripheral, accessory, or irregular vessels) should be specified, a pw-Doppler calculation of RI and PI performed, and stiffness should be estimated in comparison to the surrounding tissue. The use of quantitative SWE is currently limited by the lack of reliable cut-off values. There are no recommendations in the guidelines for the regular use of CEUS for LNs characterization. However, CEUS can be used as a supplementary technique in selected cases to assess the type of vascularization and to differentiate between necroses and melting abscesses prior to diagnostic US-guided sampling. LNs with ambiguous features on mpUS require a histological examination, and LNs likely to be metastatic with no clear assignment to a known primary tumor require a histologic diagnosis. If the LN is accessible, US-guided sampling has a high diagnostic yield at very low risk.

No single ultrasound parameter reliably distinguishes benign from malignant LN. Diagnostic confidence increases with the number of concordant abnormal findings across B-mode morphology, vascular architecture, and stiffness. Multiparametric ultrasound primarily helps to rule out malignancy and to guide targeted biopsy, rather than to replace histology.

## Figures and Tables

**Table 1:** Sonographic examination of LNs: how to perform.

Localization	Body Position and Transducer Selection	Transducer Position
Cervical	In supine position with head slightly extended backward and tilted to the opposite side. Linear transducer 9–18 MHz	The LN stations according to Som are examined using transverse and longitudinal transducer position.
Axillary	In the supine position, the arm should be slightly bent above the head. Linear transducer 2–9/9–18 MHz.	Transverse and longitudinal transducer guidance along the axillary vessels, lateral, medial, caudal, and cranial to the pectoralis minor muscle. The internal mammary LNs are examined parasternal intercostally.
Inguinal	Supine position. Linear transducer 2–9/9–18 MHz.	Examination of the LNs along the large vessels in transverse and longitudinal transducer positions.
Hepatoduodenal ligament	In supine or left-lateral position. Convex transducer 1–6 MHz	Position the hepatic hilum in the subcostal transducer position with transverse and longitudinal transducer guidance and shoulder–navel transducer position. Use deep inspiration or protrusion of the abdomen.
Paraaortal, paracaval, interaortocaval, parailiac	Supine position. Convex transducer 1–6 MHz, if sonographic imaging allows, then preferably with a linear transducer 2–9 MHz.	Examination of the LNs along the large vessels in transverse and longitudinal transducer positions.
Peripancreatic	Supine position. Convex transducer 1–6 MHz, if sonographic imaging allows, then preferably with a linear transducer 2–9 MHz.	Use protrusion of the abdomen for better visuality of the pancreas. Transversal and longitudinal transducer position.
Along the renal vessels	Supine position, convex transducer 1–6 MHz	Arms should be slightly bent above the head. Adjusting the renal hilum in the transverse section in the right and left flank
Mesenteric	Supine position. Convex transducer 1–6 MHz, preferably with a linear transducer 2–9 MHz.	Adjustment of the small and large intestine with the linear transducer. Special attention should be paid to the ileocecal region. This should be examined in transverse and longitudinal sections. Interenteric LNs should be assessed in all four quadrants. LNs should be sought in particular in areas of inflammatory or neoplastic bowel segments.
Iliac	Supine position. Convex transducer 1–6 MHz, optionally linear transducer 2–9 MHz.	Examination starting at aortic branch following along the common, external, and internal iliac vessels in transverse and longitudinal planes.

**Table 2:** Normal size of LNs in the head and neck area and other superficial LNs.

Study	Localization	Lymph Node Long-Axis Diameter (Range)	Lymph Node Short-Axis Diameters (Range)	Comments
Tschammler 1998 [44] n = 130 LNs (n = 48 benign, n = 82 malignant: metastases and lymphoma)	Cervical, submandibular, supraclavicular, inguinal LN n = 48 reactive, benign LN	*Benign*: 13.5 ± 6.0 mm (6–34 mm) *Metastases*: 19.2 ± 8.8 mm (6–43 mm) *Lymphoma*: 23.2 ± 10.5 mm (12–50 mm)	*Benign*: Thickness: 6.0 ± 2.9 mm (3–17 mm) Width: 9.1 ± 3.8 mm (4–24 mm) *Metastases*: Thickness: 11.6 ± 5.4 mm (4–29 mm) Width: 15.6 ± 7.4 mm (4–42 mm) *Lymphoma*: Thickness: 16.2 ± 9.9 mm (9–45 mm) Width: 19.4 ± 8.9 mm (10–45 mm)	Significant difference to malignant LNs (Metastases and lymphoma)
Bhatia 2012 [45] n = 55 LNs (n = 31 malignant, n = 24 benign)	Cervical LNs n = 23 reactive LNs n = 1 tuberculous LNs	N/A	*Benign*: 8.8 ± 3.4 mm (range 3–30 mm) *Malignant*: 11.4 ± 6.8 mm (range 4–30 mm)	No significant difference to malignant LNs
Ishibashi 2012 [46] N = 71 cervical LNs in oral squamous cell carcinoma (n = 31 metastatic LNs, n = 40 reactive benign LN)	n = 40 reactive LNs	N/A	*Benign*: 4.0 mm (2.4–8.3 mm) *Malignant:* 9.1 mm (3.2–19.2 mm)	
Hinz 2013 [47] N = 42 LNs (n = 21 melanoma LN metastasis, n = 21 benign)	n = 21 benign LNs cervical n = 3 (14.3%) axillary n = 3 (14.3%) inguinal n = 15 (71.4%)	*Benign*: 12.5 ± 3.66 mm *Malignant*: 19.37 ± 10.77 mm	*Benign*: 5.94 ± 1.90 mm *Malignant*: 12.30 ± 6.41 mm)	Solbiati index > 2: n = 76.21% in the benign group Solbiati index < 2: 80.9% in the malignant group.
Ghafoori 2015 [33] N = 43 patients with squamous cell carcinoma, malignant and reactive LNs	Cervical LNs n = 17 reactive LNs	*Benign*: 31.25 ± 4.82 mm *Malignant*: 34.98 ± 6.39 mm	*Benign*: 16.63 ± 5.11 mm *Malignant*: 22.62 ± 7.43 mm	Exact regional distributions of detected LNs were not specified.
Okumuş 2017 [48] Healthy probands n = 25	Cervical LNs	*Right:* 11.11 ± 3.36 mm (5.9–18 mm) *Left:* 11.8 ± 2.67 mm (7.1–16.9 mm)	*Right:* 3.56 ± 0.73 mm (2–5 mm) *Left:* 3.84 ± 0.75 mm (2.7–5.5 mm)	*Short/long-axis ratio*: *Right*: 0.34 ± 0.11 (0.21–0.64) *Left:* 0.34 ± 0.07 (0.22–0.46)
	Submandibular region	*Right:* 12.84 ± 4.4 mm (5.7–23.3 mm) *Left:* 11.94 ± 1.95 mm (7.6–16 mm)	*Right:* 3.87 ± 1.01 mm (1.8–5,8 mm) *Left:* 4.24 ± 1.07 mm (2.6–7.7 mm)	*Short/long-axis ratio:* *Right:* 0.33 ± 0.11 (0.19–0.54) *Left:* 0.36 ± 0.11 (0.2–0.66)
	Submental region	*Right:* 15.19 ± 4.62 mm (7.4–25.2 mm) *Left*: 16.18 ± 4.72 mm (9.6–29 mm)	*Right:* 4.65 ± 1.29 mm (2.4–8.8 mm) *Left:* 4.54 ± 1.28 mm (2.3–8 mm)	Short/long-axis ratio: *Right:* 0.32 ± 0.1 (0.2–0.66) *Left:* 0.29 ± 0.07 (0.18–0.48)
Lerchbaumer 2022 [49] Healthy Probands n = 34	Cervical LNs	*Benign*: 14.7 ± 6.9 mm	*Benign*: 7.4 ± 3.4 mm	*Solbiati index:* 2.11 ± 0.81
Tunçez 2023 [50] n = 100 cervical or axillary LNs, 80% were axillary	Cervical or axillary LNs in suspected malignancy, n = 36 benign LNs	N/A	*Benign:* 9.36 ± 2.93 mm	*Solbiati index:* 2.17 ± 0.59 in the benign group
Chen 2025 [42] n = 159 LNs (benign, metastatic, Lymphomas)	Cervical, axillary and inguinal. n = 37 benign LNs. The malignant LNs included LN metastases and lymphomas	20.52 ± 8.63 mm	*Benign:* 0.64 ± 3.23 mm	The benign LNs included reactive, unspecific and granulomatous lesions or tuberculosis.
He 2025 [51] n = 54 enlarged superficial LNs (benign, metastatic, lymphomas) Cervical (90%), axillary and inguinal localizations.	n = 20 benign (n = 16 reactive n = 4 tuberculous)	27 ± 7.2 mm	12 ± 6.1 mm	These were primarily enlarged LNs, including reactive, tuberculous, and malignant LNs (metastatic and lymphoma).

LN—lymph nodes.

**Table 3:** Normal size of LNs in the abdomen and retroperitoneum.

Study	Localization	Lymph Node Long-Axis Diameter (Range)	Lymph Node Short-Axis Diameter (Range)
Dietrich 1997 [17] n = 83 healthy probands and n = 20 autopsies from patients without hepatobiliary diseases.	Ventral to the portal vein Healthy probands n = 60/83 (72.3%)	13 ± 2 mm (8–17 mm)	5 ± 1 mm (0–7 mm)
	Ventral to the portal vein/autopsy study/n = 20/ultrasound	12 ± 3 mm (8–20 mm)	5 ± 1 mm (3–10 mm)
	Ventral to the portal vein/autopsy study/n = 20/macroscopically	11 ± 3 mm (8–18 mm)	5 ± 1 mm (3–8 mm)
	between portal vein and inferior vena cava Healthy probands n = 60/83 (72.3%)	14 ± 3 mm (8–20 mm)	5 ± 1 mm (3–9 mm)
	between portal vein and inferior vena cava/autopsy study/n = 20/ultrasound	13 ± 5 mm (8–20 mm)	5 ± 2 mm (3–7 mm)
	between portal vein and inferior vena cava/autopsy study/n = 20/macroscopically	12 ± 4 mm (8–19 mm)	5 ±2 mm (3–8 mm)
Dietrich 1998 [1] n = 80 healthy probands	Interaortocaval 44 /80 (55%)	11 ± 3 mm (8–18 mm)	5 ± 1 mm (3–10 mm)
	Peripancreatic 14/80 (18%)	9 ± 2 mm (8–13 mm)	5 ± 1 mm (3–7 mm).
	left aortic 32/80 (40%)	10 ± 2 mm (8–16 mm)	5 ± 1 mm (3–8 mm)
	right mesenteric 31/80 (39%)	11 ± 3 mm (8–19 mm)	5 ± 1 mm (3–9 mm)
	along the renal arteries 2/80	12 × 5 mm and 11 × 4 mm	
	Splenic hilum 0/80	No evidence of LNs in the splenic hilum.	

**Table 4:** Normal reference values of LN size according to studies.

Localization	Reference Value Long-Axis	Reference Value Short-Axis
Submandibular and in the upper cervical region (regions Ib and II) [24,27,31,32,46,48,52]		≤8 mm
Cervical regions (Ia, III, IV, V) [24,31,48]		≤5 mm
Jugulodigastric LN [23]	<30 mm	≤8 mm
Infraclavicular, internal mammary region [28]		≤4 mm
Axillary [23]		Cortical thickness < 4 mm
Hepatoduodenal ligament [17,53]	<20 mm	<8 mm
Retroperitoneal [1,53]	<30	<9 mm
Mesenterial [38]		≤5 mm
Inguinal [23]	<40 mm	Cortical thickness <2.5 mm

LN—lymph nodes.

**Table 5:** Summary of published papers with Doppler measurements of resistive index (and pulsatility index).

Study	Localization	Resistive Index (RI)	Pulsatility Index (PI)	Comments
Steinkamp 1994 [52] n = 245 cervical LNs (reactive, metastatic, lymphoma, other)	Reactive LNs n = 104	0.65 ± 0.08 Cut-off 0.8	0.92 ± 0.26 Cut-off 1.6	
Choi 1995 [74] n = 41 cervical LNs, n = 1 axillary LN, n = 1 inguinal LN	n = 19 benign LNs	0.59 ± 0.11 Cut-off < 0.8	0.90 ± 0.23 Cut-off < 1.5	Malignant LNs: RI 0.92 ± 0.23 PI 2.66 ± 1.59
Ghafoori 2015 [33] n = 43 patients with squamous cell carcinoma, metastatic and reactive LNs	Cervical LNs n = 17 reactive LNs	0.64 ± 0.08 Cut-off 0.69	1.18 ± 0.38 Cut-off 1.35	Malignant LNs: RI 0.79 ± 0.08 (*p* < 0.001) PI 1.83 ± 0.52 (*p* < 0.001)
Ying 2004 [71] n = 270 patients (metastases n = 101, lymphoma n = 21, tuberculosis n = 76, reactive n = 72?	Cervical LNs n = 72 reactive LNs	0.64 ± 0.08 Cut-off 0.7	1.05 ± 0.24 Cut-off 1.4	Tuberculosis: RI 0.71 ± 0.11

LNs—lymph nodes.

**Table 6:** Typical ultrasound criteria for lymph node characterization.

	Benign LN		Malignant LN	
	Reactive	Tuberculous	Metastatic	Lymphoma
**B-mode US**	Elongated oval shape, central hyperechoic hilum depending on location, uniform cortex width	Enlarged, rounded, hypoechoic LNs with a heterogeneous pattern, hypoechoic changes with hyperechoic margin in caseous abscesses.	Increase in short-axis and round shape, irregular widening of the cortex, hypoechoic inhomogeneities with focal metastatic infiltration. Compression/destruction of the central hilar region.	Round shape, often very large hypoechoic LNs with asymmetrical cortical widening or loss of echogenic hilum. Pronounced hypoechogenicity. LN conglomerates (bulky lesions).
**Solbiati index**	>2	variable	<2	<2
**Short/long-axis ratio**	<0.5	variable	>0.5	>0.5
**Vascularization (color Doppler imaging)**	Central vessel with regular branching; inflammation causes increased vascularization	Destroyed vascularization.	Destroyed vascularization; peripheral accessory vessels, irregular vessels.	Central and/or peripheral vessels with (irregular and pronounced) branching, high vessel density, compression of the central artery may cause necrosis.
**Doppler parameters**	RI < 0.8 PI < 1.5	RI < 0.8 PI < 1.5	RI > 0.8 PI > 1.5	No definite recommendation, often “in between”
**CEUS**	Fast and strong centrifugal enhancement, central vessel, homogeneous	Centrifugal enhancement. Melting abscesses present with hyperenhancing margins and central non-enhancing areas.	(Delayed) centripetal peripheral enhancement, non-enhanced areas in case of necroses.	Central and/or peripheral vessels, strong enhancement, in case of necrosis non-enhancing areas.
**Strain elastography**	Soft, cortex is stiffer than hilum	Stiffer compared to surrounding due to inflammation, melting caseating abscesses may be softer.	Stiffer compared to surrounding.	Stiffer compared to surrounding.

## Data Availability

Data are contained within the article. The authors refer to the complete guideline at https://www.mdpi.com/ethics#_bookmark21 (accessed on 16 March 2026).
